# DFT Approach for Predicting ^13^C NMR Shifts of Atoms Directly Coordinated to Pt: Scopes and Limitations

**DOI:** 10.3390/molecules29246052

**Published:** 2024-12-23

**Authors:** Svetlana A. Kondrashova, Shamil K. Latypov

**Affiliations:** Arbuzov Institute of Organic and Physical Chemistry, FRC Kazan Scientific Center of RAS, 420088 Kazan, Russia; kondrashovamail@gmail.com

**Keywords:** transition metals, platinum complexes, chemical shift, density functional theory, relativity

## Abstract

In this study, comparative analysis of calculated and experimental ^13^C NMR shifts for a wide range of model platinum complexes showed that, on the whole, the theory reproduces the experimental data well. The chemical shifts of carbon atoms directly bonded to Pt can be calculated well only within the framework of the fully relativistic matrix Dirac−Kohn−Sham (mDKS) level (*R*^2^ = 0.9973, *RMSE* = 3.7 ppm); however, for carbon atoms not bonded to metal, a more simple, non-relativistic approach can be used. Effective locally dense basis set schemes were developed for practical applications. The efficiency of the protocol is demonstrated using the example of the isomeric structure determination in case of several possible coordination modes.

## 1. Introduction

Platinum complexes are of great interest from the point of view of developing new drugs and various medical applications [1,2,3,4,5,6,7]. They are also important in the context of developing new catalysts, as they exhibit a catalytic effect in a number of reactions [8,9,10,11,12,13].

For the rational design of such systems and understanding of the molecular mechanism of action, it is necessary to know the precise chemical and 3D structure. Such data are especially important for solutions, since most processes occur in the liquid phase [14,15]. In this regard, modern NMR methods are very effective; two-dimensional correlation experiments, due to coupling constants, in many cases make it possible to establish the chemical structure almost directly [16]. However, fine details of the structure, such as the type of coordination with the metal, may be inaccessible or ambiguous using experiments alone [17].

To this end, NMR shifts of nuclei directly bound to the metal are very sensitive to its electronic structure and can serve as a source of information about the type of coordination and electronic structure near the metal [18,19,20,21,22,23,24,25]. This requires a theoretical approach that would allow an unambiguous and reliable connection to be established between the structure and the NMR parameters. Great progress has been made in the case of organic compounds; simple and reliable calculation protocols are proposed that allow confident predictions of ^1^H, ^13^C, ^15^N, and ^31^P NMR shifts [26,27,28,29,30,31,32,33,34,35,36,37,38]. Moreover, there are many examples where these methods have also been successfully applied to establish finer structural details such as the isomeric and tautomeric structure [39,40,41,42,43,44,45,46,47].

At the same time, for metal complexes, the achievements are noticeably more modest [18,19,21,23,48,49,50,51,52,53,54,55,56,57,58,59,60,61,62,63,64,65]. This is due to the complication of the electronic structure of systems with metals, which requires an effective method for taking into account the electron correlation effects [21,49,53]. In addition, when moving to 4*d*/5*d* metals, it may be necessary to take into account relativistic effects [22,45,46,47,48,49,50,51,52,53,54,55,56,57,64,65,66,67,68,69,70,71,72,73,74].

Only recently, it has been shown for a wide range of systems that ^13^C and ^31^P NMR shifts can be reliably calculated for most nickel complexes within the framework of fairly simple and accessible approximations [75,76,77]. Moreover, it was found that for Pd complexes, the NMR shifts of ^13^C and ^31^P can also be estimated well within the DFT approximation [78,79]. It turned out that while ^31^P shifts can be calculated within the framework of a simple, non-relativistic (NR) approach [79], for ^13^C chemical shifts, relativistic corrections can be significant [78]. For carbons with a strong covalent bond to palladium, calculations must be performed at a fully relativistic matrix Dirac−Kohn−Sham (mDKS) level [80].

Given the importance of Pt complexes in which coordination occurs via carbon, there is obvious interest in having a similar calculation tool that would allow for prediction of ^13^C NMR shifts in such systems. In this work, an attempt was made to clarify the scopes and limitations of available computational approximations for estimating ^13^C NMR shifts in platinum complexes.

## 2. Results and Discussion

To assess the quality of the quantum chemical methods, a wide range of platinum complexes (including N-C-N, P-C-P, C-C-C pincer type, based on NHC, bis-NHC, tetra-NHC, macrocyclic amine NHC, bidentate pyridyl-indolate, aliphatic, allyl, cyclooctadiene, butenone, phosphines, bis(phosphine), unsaturated phosphonium ligands, etc.) were used as models (80 complexes in total), in which at least one carbon atom participates directly in coordination with the metal (Figure S1) and for which ^13^C NMR data (132 points in total) are available (Table S1). As an initial approximation on the generalized Kohn Sham (KS) level of DFT, a combination that has proven itself well in the case of palladium complexes was used: the PBE0 functional with Pople’s basis set of double-ζ quality (6-31+G(d)) on elements for geometry optimization and the basis set of triple-ζ quality (6-311G(2d,2p)) for shielding calculations, while ECP SDD was applied to describe the metal (PBE0/{6-311G(2d,2p); Pt(SDD)}//PBE0/{6-31+G(d); Pt(SDD)}).

### 2.1. General Overview

**Figure 1:**
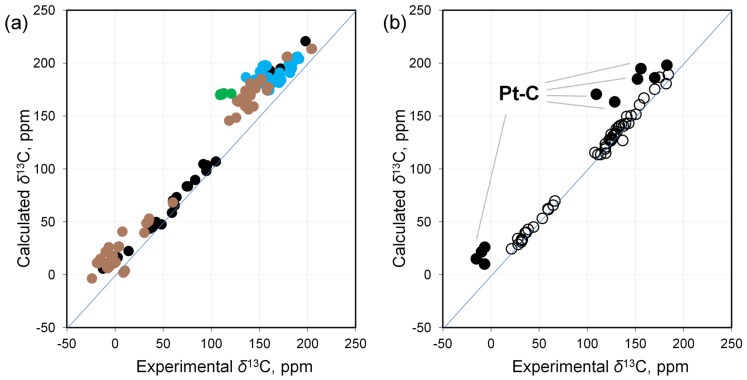
Correlation of calculated (PBE0/{6-311G(2d,2p); Pt(SDD)}//PBE0/{6-31+G(d); Pt(SDD)}) versus experimental ^13^C NMR shifts for Pt complexes: (**a**) C-Pt carbons for all title complexes (brown—carbons covalently bonded to platinum, blue—NHC carbons, green—NHC carbons in Pt(IV) complexes, black—others); (**b**) all carbons for complexes **10**–**11**, **39**, **59**, **67** (filled circles—carbon atoms directly bound to platinum, empty—all other atoms in the complex).

Overall, the calculation reproduces the experimental trends (Figure 1a, Table S1). However, it is also clear that there are noticeable deviations in some complexes.

There are strong discrepancies between the calculation and experiment for carbons covalently bound to platinum and for NHC carbons bound to metal. Even greater deviations were found for some platinum complexes in a high oxidation state. In almost all cases, the calculated shielding values are lower than the experimental ones. At the same time, for the remaining carbons not directly bound to the metal, the calculation reproduces the experimental data well (for example, for the complexes **10–11**, **39**, **59**, and **67**; Figure 1b; and Table S2). That is, the deviations from the linear correlation noted above are most likely due to the specific influence of the metal.

Theoretically, there may be several reasons for such deviations of the calculated chemical shifts (CSs) from the experimental values for C-Pt carbons: the level of theory, the flexibility of the basis sets, and the solvent effects. However, in this case, the most likely cause of such deviations may be the relativistic effects of the heavy atom (HA, platinum) on the neighboring light atom (LA, carbon), the so-called HALA effects [23,55,66,68,71,72,81]. To verify this assumption, calculations of NMR shifts were carried out within the framework of a fully relativistic matrix Dirac−Kohn−Sham (mDKS) level.

Unfortunately, calculations at this level are quite complex, and problems arose when using a fairly flexible basis on all elements. Therefore, to facilitate calculations, the locally dense basis sets (LDBS) scheme [82,83,84,85,86] was applied: large triple-ζ quality basis sets (ucc-pVTZ) on spectator atoms and vicinal atoms to metal and significantly smaller double-ζ quality basis sets (ucc-pVDZ) on the remaining atoms (denoted as “TZ_DZ”). However, even such a pruned version of the LDBS turned out to be unaffordable for calculations for some relatively heavy complexes (e.g., **1**–**2**, **28**–**34**, **77**–**80**). For them, a further compromise had to be made; in the immediate vicinity of the metal and spectator atoms, triple-ζ quality basis sets (ucc-pVTZ) were used; while for the next layer, the double-ζ quality basis sets (ucc-pVDZ) were applied; and then for the entire periphery, a reduced unpolarized Jensen basis set (upc-0) was used (denoted as “TZ_DZ_UPC”, see details in the experimental section).

To test the suitability of such a scheme, calculations were carried out for a number of systems (**3**, **4**, **19**, **68**) using these two LDBSs. It turned out that the difference in the calculation results for C-Pt carbons using “TZ_DZ” and “TZ_DZ_UPC” LDBSs is indeed small: ~2 ppm (Table S3). Thus, this approximation seems to be adequate, reflects relativistic effects, and becomes feasible for relatively large systems. This basis sets were used for all remaining heavy systems.

**Figure 2:**
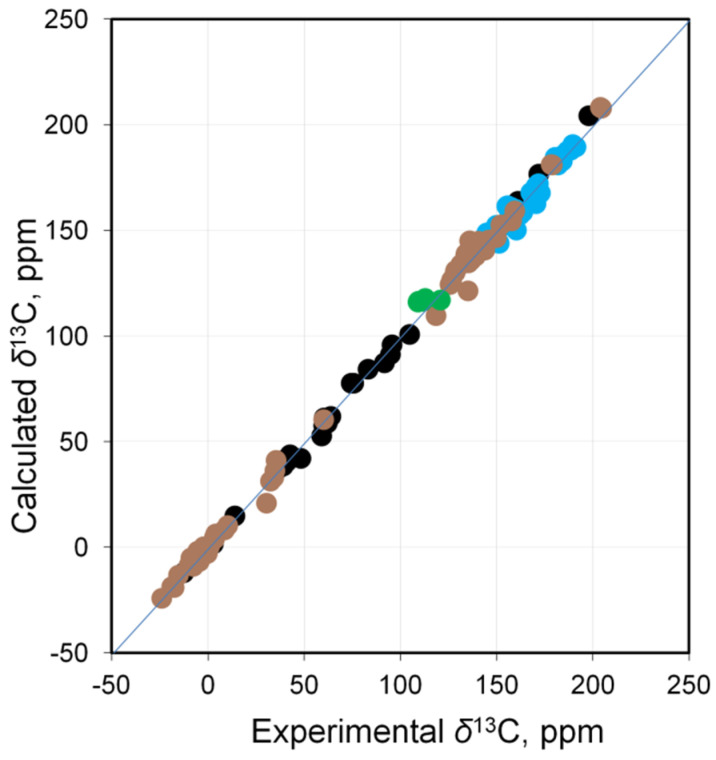
Correlation of calculated (mDKS level) versus experimental ^13^C NMR shifts for all Pt complexes (see Figure 1 for color explanation).

In general, it can be stated that there is a significant improvement in the correlation of calculated values versus experimental ones (*R*^2^ = 0.9973 versus 0.9697, Table S1). This is especially evident for NHC carbons, as well as for carbons covalently bonded to the metal, particularly in Pt(IV) complexes (Figure 2).

Thus, the calculations of the CSs of carbons directly bonded to platinum within the fully relativistic matrix Dirac−Kohn−Sham level for a wide range of systems reproduce the main trends well, and a good correlation with the experiment is observed. The only problem that remains is the presence of a systematic error in the calculated NMR shifts inherent in DFT methods [30], which increases in lower fields, i.e., as it moves away from the reference.

Such errors can be corrected by the linear scaling procedure [87,88] according to Equation (1) using the regression analysis parameters for all sets (Table S4).

*δ*_scaled_ = (*δ* − Intercept)/Slope, (1)

where *δ* is the calculated CS for a particular nucleus, and the intercept (−0.9) and slope (1.06) are scaling factors. The intercept fixes the error for a reference compound, while the slope helps to correct the systematic errors. Thus, the application of this procedure allows for the evaluation of ^13^C CSs in Pt complexes with *RMSE* = 3.7 ppm.

It is worth noting that the use of a similar procedure for calculation at the KS level leads to a value of *RMSE* = 12.6 ppm, which is several times higher than the errors when using the mDKS level.

### 2.2. An Attempt to Improve Computational Protocol

Having confirmed that the ^13^C NMR shifts of C-Pt carbon could be calculated reasonably well, we tried to find out whether the agreement between the calculation and experiment could be improved. Unfortunately, in this case, due to the complexity of the main stage—the shielding calculation at the mDKS level—there are few opportunities for varying the parameters. However, we tried to evaluate how the calculation approximation at the optimization stage can affect the CSs. To assess the quality of the calculations, a “training” set of 23 complexes representing the main types and typical problems was selected (**1**–**3**, **8**, **19**–**20**, **28**, **31**, **35**, **38**–**39**, **53**, **59**–**61**, **64**–**65**, **68**, **75**, **77**–**80**).

First, we tried to augment the basis sets at the geometry optimization stage. Previously, for nickel complexes, we found that the use of a more flexible basis set at this stage improves the results of calculations [75]. Therefore, in this case, a more flexible basis set 6-311+G(2d) was used on the elements, while on the metal ECP SDD was used (PBE0/{6-311+G(2d); Pt(SDD)}). The shielding calculation was carried out at the same level as above ((PBE0/{6-311G(2d); Pt(SDD)})). It turned out that calculations with this combination at a non-relativistic (NR) level led to only minimal changes both quantitatively and qualitatively (*R*^2^ = 0.9654 versus 0.9651, Table S5).

The calculation of the shielding at a fully relativistic level using this geometry leads to an improvement in correlation compared to the NR approximation (*R*^2^ = 0.9979). However, if we compare these results with similar calculations using the original geometry (PBE0/{6-31+G(d); Pt(SDD)}), the difference is a even little worse (*R*^2^ = 0.9979 versus 0.9984, Table S5). Thus, it seems that changes in the basis sets at the geometry optimization stage do not lead to a noticeable change in the results.

Secondly, an attempt was made to take into account medium effects at the geometry optimization stage. The calculation was carried out within the framework of the PCM model for the solvents used in the experiment, while the basis sets remained the same as at the initial stage ((PBE0/{6-311G(2d,2p); Pt(SDD)}//PBE0/{6-31+G(d); Pt(SDD)}). It turned out that calculations at an NR level with these parameters lead to only minimal improvements (*R*^2^ = 0.9688 versus 0.9651, Table S5). It can be noted that some systematic increase in shielding is predicted only for NHC carbons, while for the rest, the changes are significantly smaller and in different directions.

Shielding calculations at the mDKS level using this geometry also show an improvement in correlation compared to the NR level (*R*^2^ = 0.9987 versus 0.9688, Table S5). However, if we compare these calculations with similar ones on the geometry obtained without taking into account the solvent effect, the changes are minimal (*R*^2^ = 0.9987 versus 0.9984, Table S5). That is, the solvent effect also does not change the overall picture. Therefore, if we take into account that geometry optimization with a solvent is more resource-intensive or sometimes convergence problems arise, then there is no point in complicating the calculation protocol.

Finally, we tried to use another available computational approximation for the shielding calculation using the original geometry (PBE0/{6-31+G(d); Pt(SDD)}), which showed itself to be quite good in relativistic calculations. Namely, NMR-DKH (TZ2P) basis sets were used on Pt which were partially contracted as a triple-zeta doubly polarized scheme with all coefficients obtained from a Douglas–Kroll–Hess second-order scalar relativistic calculation [89]. It turned out that in this case, there is a slight improvement compared to the initial NR approximation (*R*^2^ = 0.9723 versus 0.9651, Table S5). However, this protocol does not allow us to correct those problems associated with the influence of spin-orbit (SO) relativistic effects.

Thus, we came to the conclusion that the original relativistic approximation is optimal from the point of view of “price-and-quality”.

### 2.3. From C-Pt NMR Shifts to Isomeric Structure

**Figure 3:**
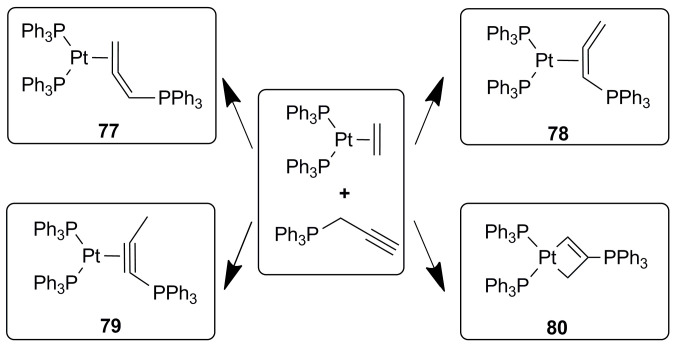
Structures of possible reaction products of the propargylic salt [Ph_3_PCH_2_C≡CH]PF_6_ with [Pt(C_2_H_4_)(PPh_3_)_2_].

Having shown that the ^13^C NMR shifts of carbon atoms directly bonded to platinum can be predicted with fairly good accuracy, we demonstrate how this tool can be used in fine structural analysis. As an example, we consider the identification of the isomeric structure of complexes that can be obtained in the reaction of the propargylic salt [Ph_3_PCH_2_C≡CH]PF_6_ with [Pt(C_2_H_4_)(PPh_3_)_2_] (Figure 3). Firstly, the result of the reaction may be the *η*^2^-allenylphosphonium salt [Pt(*η*^2^-CH_2_=C=CHPPh_3_)-(PPh_3_)_2_]PF_6_ (**77**) via a metal-mediated propargylic rearrangement, where the allenylphosphonium ligand is coordinated through the terminal C=C group. Secondly, isomerization of the complex may occur in the solution to generate the salt [Pt{*η*^2^-C(PPh_3_)=C=CH_2_}(PPh_3_)_2_]PF_6_ (**78**), wherein the allene is coordinated by the internal C=C bond. Thirdly, according to calculations [90], the isomerization of the propargylic cation [Ph_3_PCH_2_C≡CH]^+^ to the *α*-alkynyl isomer [Ph_3_PC≡CCH_3_]^+^ is thermodynamically favorable; therefore, the reaction product with this ligand is also quite expected (**79**). Finally, potentially, the thermal isomerization of [6]PF6 (**77**) into the *κ*^2^C,C′ isomer, [Pt{*κ*^2^-CH=C(PPh_3_)CH_2_}(PPh_3_)_2_]BF_4_ (**80**)), cannot be ruled out [90].

In this particular case, the presence of additional spin labels in the form of NMR active phosphorus and protons makes it possible to obtain independent additional information about the isomeric structure of the ligand fragment in the solution and, thus, obtain reliably assigned ^13^C NMR data for a whole series of isomers. In our analysis, we will not take this information into account and will appeal only to ^13^C NMR shifts for comparative analysis. Thus, for three carbon nuclei of the ligand, we have four options for bonding with the metal. For each of the variants, there is a set of experimental ^13^C CSs, which, although markedly different from each other (Table 1), are initially difficult to assign to any particular structure of the complex simply based on some intuitive considerations. For example, for C1, the CS is in all variants in fairly low fields, while for C3, it is in high fields. At the same time, for C2, the changes are greater; although in variants **a**, **b**, and **c**, the difference is only in increments of 10 ppm.

However, calculations for each of the hypotheses and comparisons with the experimental data for each of the isomers allow us to clearly identify the correct hypothesis and reject the incorrect one (Table 1). Namely, only in the case of a correct hypothesis, the difference between the experimental and calculated CSs for all three carbons is, in most cases, less than 5 ppm. At the same time, for the incorrect option, these deviations are several times higher for at least one or two carbon atoms at a time (Table 1). Thus, the calculation of ^13^C NMR shifts for various coordination modes and comparison with experimental data makes it possible to reliably identify the fine structure of the Pt complex.

## 3. Materials and Methods

### Calculations

Non-relativistic quantum chemical calculations were carried out within the framework of the generalized Kohn Sham (KS) density functional theory [91] with the Gaussian 16 [92] (Revision A.03) software packages using PBE0 [93] functional and Pople’s basis sets [94,95,96,97,98,99,100,101]. For the Pt center, the quasi-relativistic Stuttgart–Dresden ECP60MWB was used with the corresponding (8s7p6d)/(6s5p3d) GTO valence basis set [102] (denoted as “SDD”) and NMR-DKH (TZ2P) basis set [89]. Wherever possible, geometry optimization was started from an X-ray structure. For most of the complexes, the calculations were carried out for all possible conformers/isomers, and results for the lowest energy forms were used in the analysis. To take into account the medium effects, calculations were carried out in the framework of the Polarizable Continuum Model [103] (denoted as “PCM”) with the same solvent as that used in NMR experiments. ^13^C NMR shifts were calculated by the GIAO method [104]. All ^13^C data were referenced to tetramethylsilane (TMS), which was calculated under the same conditions.

Fully relativistic DFT ^13^C NMR shifts calculations have been carried out at the matrix Dirac-Kohn-Sham (mDKS) level [80] with the ReSpect-MAG code [105]. The four-component mDKS calculations were conducted with PBE0 functional. The uncontracted Dyall valence double-ζ basis set [106] was used for the Pt center. For ligand atoms, two locally dense basis sets (LDBS) schemes [82,83,84,85,86] were used: (1) Dunning’s triple-ζ quality basis sets (ucc-pVTZ) on spectator atoms and atoms vicinal to Pt center, and double-ζ quality basis sets (ucc-pVDZ) [107,108,109] were applied on the remaining atoms (denoted as “TZ_DZ”); (2) Dunning’s triple-ζ quality basis sets (ucc-pVTZ) on spectator atoms and atoms vicinal to Pt center, double-ζ quality basis sets (ucc-pVDZ) on the next layer and unpolarized Jensen basis set (upc-0) [110,111,112,113] on the remaining atoms were applied (denoted as “TZ_DZ_UPC”). For relativistic shielding calculations, the PBE0/{6-31+G(d); Pt(SDD)} geometry were used unless otherwise stated.

The NMR-DKH (TZ2P) basis sets were downloaded from the EMSL basis set library for the Gaussian package [114,115,116].

## 4. Conclusions

Comparative analysis of calculated (GIAO method, KS and mDKS levels) and experimental ^13^C NMR shifts for a wide range of diamagnetic Pt complexes was carried out. Several different approximations were tested.

On the whole, the CSs of carbon atoms directly bonded to Pt can be well-calculated only within the framework of the fully relativistic matrix Dirac−Kohn−Sham (mDKS) level (*R*^2^ = 0.9973, *RMSE* = 3.7 ppm). At the same time, the CSs of carbon atoms not directly bonded to Pt can be calculated within the framework of more a simple, non-relativistic approach. Effective, locally dense basis set schemes were developed for practical applications.

To sum it up, the PBE0/{6-31+G(d); Pd(SDD)} approximation can be recommended for geometry optimization. For the shielding calculation of C-Pt carbons, a fully relativistic matrix Dirac−Kohn−Sham (mDKS) level has to be used with corresponding locally dense basis sets. For the rest of the carbon atoms, the calculations at the KS level (PBE0/{6-311G(2d,2p); Pt(SDD)}) provide reasonable ^13^C NMR shifts.

The efficiency of the protocol is demonstrated using the example of determining an isomeric structure in case of multiple coordination modes.

## Figures and Tables

**Table 1:** Experimental and calculated ^13^C CSs for ligand carbons of complexes **77**–**80** and difference between them for different variants of assignment.

Complexes	Exp. ^13^C Shifts	Calculated ^113^C Shifts			
		**77**	**78**	**79**	**80**
		204.3	155.9	176.7	163.8
		73.9	106.2	87.4	126.1
		14.7	1.6	9.2	−12.2
	197.9	6.4	−42.0	−21.2	−34.1
**77**	83.3	−9.4	22.9	4.1	42.8
	14.0	0.7	−12.4	−4.8	−26.2
	158.4	45.9	−2.5	18.3	5.4
**78**	103.7	−29.8	2.5	−16.3	22.4
	2.8	11.9	−1.2	6.4	−15.0
	172.0	32.3	−16.1	4.7	−8.2
**79**	91.7	−17.8	14.5	−4.3	34.4
	12.3	2.4	−10.7	−3.1	−24.5
	161.1	43.2	−5.2	15.6	2.7
**80**	136.3	−62.4	−30.1	−48.9	−9.8
	−12.6	27.3	14.2	21.8	0.4

^1^mDKS/TZ_DZ_UPC, scaled. Cases with small differences between experimental and calculated ^13^C CSs colored in green.

## Data Availability

All data are contained within the article and Supporting Information.

## Supplementary Materials

The following supporting information can be downloaded at: https://www.mdpi.com/article/10.3390/molecules29246052/s1, Figure S1: Model Pt complexes; Table S1: Experimental, calculated ^13^C NMR shifts (ppm) for all model Pt complexes **1**–**80** [117,118,119,120,121,122,123,124,125,126,127,128,129,130,131,132,133,134,135,136,137,138,139,140]; Table S2: Experimental and calculated ^13^C NMR shifts (ppm) for all carbon atoms in complexes **10**, **11**, **39**, **59** and **67**; Table S3: Experimental and calculated (using three different levels)^13^C NMR shifts (ppm) for complexes **19**, **20**, **57** and **68**; Table S4: Empirical scaling factors obtained by the linear regression analysis of calculated and experimental *δ*^13^C NMR shifts for all model complexes; Table S5: Experimental and calculated ^13^C NMR shifts (ppm) for “training” set of Pt complexes (**1–3**, **8**, **19**–**20**, **28**, **31**, **35**, **38**–**39**, **53**, **59**–**61**, **64**–**65**, **68**, **75**, **77**–**80**).

## References

[1] Wang X., Guo Z. (2013). Targeting and Delivery of Platinum-Based Anticancer Drugs. Chem. Soc. Rev..

[2] Xu Z., Wang Z., Deng Z., Zhu G. (2021). Recent Advances in the Synthesis, Stability, and Activation of Platinum(IV) Anticancer Prodrugs. Coord. Chem. Rev..

[3] Tuo W., Xu Y., Fan Y., Li J., Qiu M., Xiong X., Li X., Sun Y. (2021). Biomedical Applications of Pt(II) Metallacycle/Metallacage-Based Agents: From Mono-Chemotherapy to Versatile Imaging Contrasts and Theranostic Platforms. Coord. Chem. Rev..

[4] De Castro F., De Luca E., Benedetti M., Fanizzi F.P. (2022). Platinum Compounds as Potential Antiviral Agents. Coord. Chem. Rev..

[5] Paprocka R., Wiese-Szadkowska M., Janciauskiene S., Kosmalski T., Kulik M., Helmin-Basa A. (2022). Latest Developments in Metal Complexes as Anticancer Agents. Coord. Chem. Rev..

[6] Lippert B., Sanz Miguel P.J. (2022). Beyond Sole Models for the First Steps of Pt-DNA Interactions: Fundamental Properties of Mono(Nucleobase) Adducts of Pt^II^ Coordination Compounds. Coord. Chem. Rev..

[7] Seah J.W.K., Lee J.X.T., Li Y., Pullarkat S.A., Tan N.S., Leung P.-H. (2021). Chelating Phosphine–N-Heterocyclic Carbene Platinum Complexes via Catalytic Asymmetric Hydrophosphination and Their Cytotoxicity Toward MKN74 and MCF7 Cancer Cell Lines. Inorg. Chem..

[8] Konnick M.M., Bischof S.M., Yousufuddin M., Hashiguchi B.G., Ess D.H., Periana R.A. (2014). A Mechanistic Change Results in 100 Times Faster CH Functionalization for Ethane versus Methane by a Homogeneous Pt Catalyst. J. Am. Chem. Soc..

[9] Bonnington K.J., Zhang F., Moustafa M.M.A.R., Cooper B.F.T., Jennings M.C., Puddephatt R.J. (2011). Activation of Anisole by Organoplatinum(II) Complexes: Evidence for Rate-Determining C–H Activation. Organometallics.

[10] Hughes A.E., Haque N., Northey S.A., Giddey S. (2021). Platinum Group Metals: A Review of Resources, Production and Usage with a Focus on Catalysts. Resources.

[11] Clarke M.L. (2001). Recent Advances in Homogeneous Catalysis Using Platinum Complexes. Polyhedron.

[12] Ren X., Wang Y., Liu A., Zhang Z., Lv Q., Liu B. (2020). Current Progress and Performance Improvement of Pt/C Catalysts for Fuel Cells. J. Mater. Chem. A.

[13] Keyes L., Wang T., Patrick B.O., Love J.A. (2012). Pt Mediated C–H Activation: Formation of a Six Membered Platinacycle via C*sp*^3^-H Activation. Inorg. Chim. Acta.

[14] Liu T., Wang X., Hoffmann C., DuBois D.L., Bullock R.M. (2014). Heterolytic Cleavage of Hydrogen by an Iron Hydrogenase Model: An Fe-H⋅⋅⋅H-N Dihydrogen Bond Characterized by Neutron Diffraction. Angew. Chem. Int. Ed..

[15] Yang J.Y., Smith S.E., Liu T., Dougherty W.G., Hoffert W.A., Kassel W.S., DuBois M.R., DuBois D.L., Bullock R.M. (2013). Two Pathways for Electrocatalytic Oxidation of Hydrogen by a Nickel Bis(Diphosphine) Complex with Pendant Amines in the Second Coordination Sphere. J. Am. Chem. Soc..

[16] Derome A.E. (1988). Modern NMR Techniques for Chemistry Research.

[17] Latypov S.K., Ganushevich Y.S., Kondrashova S.A., Kharlamov S.V., Milyukov V.A., Sinyashin O.G. (2018). Structural diversity and dynamics of nickel complexes with ambidentate phosphorus heterocycles. Organometallics.

[18] Gordon C.P., Raynaud C., Andersen R.A., Copéret C., Eisenstein O. (2019). Carbon-13 NMR Chemical Shift: A Descriptor for Electronic Structure and Reactivity of Organometallic Compounds. Acc. Chem. Res..

[19] Halbert S., Copéret C., Raynaud C., Eisenstein O. (2016). Elucidating the link between NMR chemical shifts and electronic structure in d^0^ olefin metathesis catalysts. J. Am. Chem. Soc..

[20] Rubin M., Trofimov A., Gevorgyan V. (2005). Can polarization of triple bond in tolanes be deduced from ^13^C NMR shifts? Re-evaluation of factors affecting regiochemistry of the palladium-catalyzed hydrostannation of alkynes. J. Am. Chem. Soc..

[21] Buhl M., Kaupp M., Malkina O.L., Malkin V.G. (1999). The DFT route to NMR chemical shifts. J. Comput. Chem..

[22] Greif A.H., Hrobárik P., Kaupp M. (2017). Insights into *trans*-Ligand and Spin-Orbit Effects on Electronic Structure and Ligand NMR Shifts in Transition-Metal Complexes. Chem. Eur. J..

[23] Vícha J., Straka M., Munzarova M.L., Marek R. (2014). Mechanism of spin—Orbit effects on the ligand NMR chemical shift in transition-metal complexes: Linking NMR to EPR. J. Chem. Theory Comput..

[24] Pazderski L., Webb G. (2013). ^15^N and ^31^P NMR Coordination Shifts in Transition Metal Complexes with Nitrogen- and Phosphorus-Containing Heterocycles. Annual Reports on NMR Spectroscopy.

[25] Pazderski L., Toušek J., Sitkowski J., Kozerski L., Szłyk E. (2009). ^1^H, ^13^C and ^15^N nuclear magnetic resonance coordination shifts in Au(III), Pd(II) and Pt(II) chloride complexes with phenylpyridines. Magn. Reson. Chem..

[26] Bagno A., Rastrelli F., Saielli G. (2007). Prediction of the ^1^H and ^13^C NMR Spectra of *α*-D-Glucose in Water by DFT Methods and MD Simulations. J. Org. Chem..

[27] Rosselli S., Bruno M., Maggio A., Bellone G., Formisano C., Mattia C.A., Di Micco S., Bifulco G. (2007). Two New Flavonoids from Bonannia Graeca: A DFT-NMR Combined Approach in Solving Structures. Eur. J. Org. Chem..

[28] Bifulco G., Riccio R., Gaeta C., Neri P. (2007). Quantum Mechanical Calculations of Conformationally Relevant ^1^H and ^13^C NMR Chemical Shifts of N-, O-, and S-Substituted Calixarene Systems. Chem. Eur. J..

[29] Balandina A., Kalinin A., Mamedov V., Figadère B., Latypov S. (2005). Structure-NMR Chemical Shift Relationships for Novel Functionalized Derivatives of Quinoxalines. Magn. Reson. Chem..

[30] Lodewyk M.W., Siebert M.R., Tantillo D.J. (2011). Computational Prediction of ^1^H and ^13^C Chemical Shifts: A Useful Tool for Natural Product, Mechanistic, and Synthetic Organic Chemistry. Chem. Rev..

[31] Chimichi S., Boccalini M., Matteucci A., Kharlamov S.V., Latypov S.K., Sinyashin O.G. (2010). GIAO DFT ^13^C/^15^N Chemical Shifts in Regioisomeric Structure Determination of Fused Pyrazoles. Magn. Reson. Chem..

[32] Flaig D., Maurer M., Hanni M., Braunger K., Kick L., Thubauville M., Ochsenfeld C. (2014). Benchmarking hydrogen and carbon NMR chemical shifts at HF, DFT, and MP2 levels. J. Chem. Theory Comput..

[33] Semenov V.A., Krivdin L.B. (2020). DFT computational schemes for ^1^H and ^13^C NMR chemical shifts of natural products, exemplified by strychnine. Magn. Reson. Chem..

[34] Iron M.A. (2017). Evaluation of the Factors Impacting the Accuracy of ^13^C NMR Chemical Shift Predictions using Density Functional Theory—The Advantage of Long-Range Corrected Functionals. J. Chem. Theory Comput..

[35] Hoffmann F., Li D.-W., Sebastiani D., Brüschweiler R. (2017). Improved quantum chemical NMR chemical shift prediction of metabolites in aqueous solution toward the validation of unknowns. J. Phys. Chem. A.

[36] Potmischil F., Hillebrand M., Kalchhauser H. (2020). Hydroacridines: Part 33. An experimental and DFT study of the ^13^C NMR chemical shifts of the nitrosamines derived from the six stereoisomers of tetradecahydroacridine. Magn. Reson. Chem..

[37] Latypov S.K., Polyancev F.M., Yakhvarov D.G., Sinyashin O.G. (2015). Quantum chemical calculations of ^31^P NMR chemical shifts: Scopes and limitations. Phys. Chem. Chem. Phys..

[38] Xin D., Sader C.A., Chaudhary O., Jones P.J., Wagner K., Tautermann C.S., Yang Z., Busacca C.A., Saraceno R.A., Fandrick K.R. (2017). Development of a ^13^C NMR chemical shift prediction procedure using B3LYP/cc-pVDZ and empirically derived systematic error correction terms: A computational small molecule structure elucidation method. J. Org. Chem..

[39] Claramunt R.M., López C., Santa María M.D., Sanz D., Elguero J. (2006). The Use of NMR Spectroscopy to Study Tautomerism. Prog. Nucl. Magn. Reson. Spectrosc..

[40] Bifulco G., Dambruoso P., Gomez-Paloma L., Riccio R. (2007). Determination of Relative Configuration in Organic Compounds by NMR Spectroscopy and Computational Methods. Chem. Rev..

[41] Latypov S., Balandina A., Boccalini M., Matteucci A., Usachev K., Chimichi S. (2008). Structure Determination of Regioisomeric Fused Heterocycles by the Combined Use of 2D NMR Experiments and GIAO DFT ^13^C Chemical Shifts. Eur. J. Org. Chem..

[42] Di Micco S., Chini M.G., Riccio R., Bifulco G. (2010). Quantum Mechanical Calculation of NMR Parameters in the Stereostructural Determination of Natural Products. Eur. J. Org. Chem..

[43] Navarro-Vázquez A. (2017). State of the art and perspectives in the application of quantum chemical prediction of ^1^H and ^13^C chemical shifts and scalar couplings for structural elucidation of organic compounds. Magn. Reson. Chem..

[44] Bagno A., Saielli G. (2015). Addressing the stereochemistry of complex organic molecules by density functional theory-NMR. Wiley Interdiscip. Rev. Comput. Mol. Sci..

[45] Grimblat N., Sarotti A.M. (2016). Computational chemistry to the rescue: Modern toolboxes for the assignment of complex molecules by GIAO NMR calculations. Chem. Eur. J..

[46] Tantillo D.J. (2013). Walking in the woods with quantum chemistry—Applications of quantum chemical calculations in natural products research. Nat. Prod. Rep..

[47] Toukach F.V., Ananikov V.P. (2013). Recent advances in computational predictions of NMR parameters for the structure elucidation of carbohydrates: Methods and limitations. Chem. Soc. Rev..

[48] Kaupp M., Malkin V.G., Malkina O.L., Salahub D.R. (1995). Calculation of ligand NMR chemical shifts in transition-metal complexes using ab initio effective-core potentials and density functional theory. Chem. Phys. Lett..

[49] Kaupp M., Malkina O.L., Malkin V.G. (1997). The calculation of ^17^O chemical shielding in transition metal oxo complexes. I. Comparison of DFT and ab initio approaches, and mechanisms of relativity-induced shielding. J. Chem. Phys..

[50] Del Rosal I., Maron L., Poteau R., Jolibois F. (2008). DFT calculations of ^1^H and ^13^C NMR chemical shifts in transition metal hydrides. Dalton Trans..

[51] Kaupp M., Malkin V.G., Malkina O.L., Salahub D.R. (1995). Scalar relativistic effects on ^17^O NMR chemical shifts in transition-metal oxo complexes. An ab initio ECP/DFT study. J. Am. Chem. Soc..

[52] Davidson E.R. (2000). Computational transition metal chemistry. Chem. Rev..

[53] Autschbach J., Ziegler T. (2003). Double perturbation theory: A powerful tool in computational coordination chemistry. Coord. Chem. Rev..

[54] Pawlak T., Munzarová M.L., Pazderski L., Marek R. (2011). Validation of relativistic DFT approaches to the calculation of NMR chemical shifts in square-planar Pt^2+^ and Au^3+^ complexes. J. Chem. Theory Comput..

[55] Vícha J., Novotny J., Straka M., Repisky M., Ruud K., Komorovsky S., Marek R. (2015). Structure, solvent, and relativistic effects on the NMR chemical shifts in square-planar transition-metal complexes: Assessment of DFT approaches. Phys. Chem. Chem. Phys..

[56] Bagno A., Saielli G. (2011). Relativistic DFT calculations of the NMR properties and reactivity of transition metal methane σ-complexes: Insights on C−H bond activation. Phys. Chem. Chem. Phys..

[57] Autschbach J. (2004). The Calculation of NMR Parameters in Transition Metal Complexes. Principles and Applications of Density Functional Theory in Inorganic Chemistry I.

[58] Krykunov M., Ziegler T., van Lenthe E. (2009). Implementation of a Hybrid DFT Method for Calculating NMR Shieldings Using Slater-Type Orbitals with Spin—Orbital Coupling Included. Applications to ^187^Os, ^195^Pt, and ^13^C in Heavy-Metal Complexes. J. Phys. Chem. A.

[59] Termaten A.T., Aktas H., Schakel M., Ehlers A.W., Lutz M., Spek A.L., Lammertsma K. (2003). Terminal Phosphinidene Complexes Cp^R^(L)M=PAr of the Group 9 Transition Metals Cobalt, Rhodium, and Iridium. Synthesis, Structures, and Properties. Organometallics.

[60] Vaara J., Malkina O.L., Stoll H., Malkin V.G., Kaupp M. (2001). Study of relativistic effects on nuclear shieldings using density-functional theory and spin—Orbit pseudopotentials. J. Chem. Phys..

[61] Ehlers A.W., Ruiz-Morales Y., Baerends E.J., Ziegler T. (1997). Dissociation Energies, Vibrational Frequencies, and ^13^C NMR Chemical Shifts of the 18-Electron Species [M(CO)_6_]^n^ (M = Hf-Ir, Mo, Tc, Ru, Cr, Mn, Fe). A Density Functional Study. Inorg. Chem..

[62] Zurek E., Ziegler T. (2002). Toward the identification of dormant and active species in MAO (methylaluminoxane)-activated, dimethylzir- conocene-catalyzed olefin polymerization. Organometallics.

[63] Gracia J., Martín A., Mena M., Morales-Varela M.D.C., Poblet J.M., Santamaría C. (2003). Intercalation of alkali metal cations into layered organotitanium oxides. Angew. Chem. Int. Ed..

[64] Vícha J., Patzschke M., Marek R. (2013). A Relativistic DFT Methodology for Calculating the Structures and NMR Chemical Shifts of Octahedral Platinum and Iridium Complexes. Phys. Chem. Chem. Phys..

[65] Das A., Das U., Das A.K. (2023). Relativistic Effects on the Chemical Bonding Properties of the Heavier Elements and Their Compounds. Coord. Chem. Rev..

[66] Vícha J., Novotny J., Komorovsky S., Straka M., Kaupp M., Marek R. (2020). Relativistic heavy-neighbor-atom effects on NMR shifts: Concepts and trends across the periodic table. Chem. Rev..

[67] Autschbach J., Zheng S., Webb G. (2009). Relativistic computations of NMR parameters from first principles: Theory and applications. Annual Reports on NMR Spectroscopy.

[68] Hrobárik P., Hrobáriková V., Meier F., Repiský M., Komorovský S., Kaupp M. (2011). Relativistic four-component DFT calculations of ^1^H NMR chemical shifts in transition-metal hydride complexes: Unusual high-field shifts beyond the Buckingham—Stephens model. J. Phys. Chem. A.

[69] Vícha J., Komorovsky S., Repisky M., Marek R., Straka M. (2018). Relativistic spin—Orbit heavy atom on the light atom nmr chemical shifts: General trends across the periodic table explained. J. Chem. Theory Comput..

[70] Novotny J., Vícha J., Bora P.L., Repisky M., Straka M., Komorovsky S., Marek R. (2017). Linking the character of the metal—Ligand bond to the ligand NMR shielding in transition-metal complexes: NMR contributions from spin—Orbit coupling. J. Chem. Theory Comput..

[71] Greif A.H., Hrobárik P., Hrobáriková V., Arbuznikov A.V., Autschbach J., Kaupp M. (2015). A relativistic quantum-chemical analysis of the trans influence on 1H NMR hydride shifts in square-planar platinum(II) complexes. Inorg. Chem..

[72] Vícha J., Foroutan-Nejad C., Pawlak T., Munzarová M.L., Straka M., Marek R. (2015). Understanding the electronic factors responsible for ligand spin—Orbit NMR shielding in transition-metal complexes. J. Chem. Theory Comput..

[73] Castro A.C., Balcells D., Repisky M., Helgaker T., Cascella M. (2020). First-Principles Calculation of ^1^H NMR Chemical Shifts of Complex Metal Polyhydrides: The Essential Inclusion of Relativity and Dynamics. Inorg. Chem..

[74] Castro A.C., Fliegl H., Cascella M., Helgaker T., Repisky M., Komorovsky S., Medrano M.Á., Quiroga A.G., Swart M. (2019). Four-Component Relativistic ^31^P NMR Calculations for Trans-Platinum(II) Complexes: Importance of the Solvent and Dynamics in Spectral Simulations. Dalton Trans..

[75] Kondrashova S.A., Polyancev F.M., Ganushevich Y.S., Latypov S.K. (2021). DFT approach for predicting ^13^C NMR shifts of atoms directly coordinated to nickel. Organometallics.

[76] Latypov S.K., Kondrashova S.A., Polyancev F.M., Sinyashin O.G. (2020). Quantum chemical calculations of ^31^P NMR chemical shifts in nickel complexes: Scope and limitations. Organometallics.

[77] Payard P.A., Perego L.A., Grimaud L., Ciofini I. (2020). A DFT protocol for the prediction of ^31^P NMR chemical shifts of phosphine ligands in first-row transition-metal complexes. Organometallics.

[78] Kondrashova S.A., Latypov S.K. (2023). DFT Approach for Predicting ^13^C NMR Shifts of Atoms Directly Coordinated to Pd. Organometallics.

[79] Kondrashova S.A., Polyancev F.M., Latypov S.K. (2022). DFT Calculations of ^31^P NMR Chemical Shifts in Palladium Complexes. Molecules.

[80] Komorovský S., Repiský M., Malkina O.L., Malkin V.G., Malkin Ondík I., Kaupp M. (2008). A Fully Relativistic Method for Calculation of Nuclear Magnetic Shielding Tensors with a Restricted Magnetically Balanced Basis in the Framework of the Matrix Dirac–Kohn–Sham Equation. J. Chem. Phys..

[81] Kaupp M., Schwerdtfeger P. (2004). Relativistic Effects on NMR Chemical Shifts. Theoretical and Computational Chemistry.

[82] Chesnut D.B., Moore K.D. (1989). Locally Dense Basis Sets for Chemical Shift Calculations. J. Comput. Chem..

[83] Chesnut D.B., Rusiloski B.E., Moore K.D., Egolf D.A. (1993). Use of Locally Dense Basis Sets for Nuclear Magnetic Resonance Shielding Calculations. J. Comput. Chem..

[84] Chesnut D.B., Byrd E.F.C. (1996). The Use of Locally Dense Basis Sets in Correlated NMR Chemical Shielding Calculations. Chem. Phys..

[85] Provasi P.F., Aucar G.A., Sauer S.P.A. (2000). The Use of Locally Dense Basis Sets in the Calculation of Indirect Nuclear Spin–Spin Coupling Constants: The Vicinal Coupling Constants in H_3_C–CH_2_X (X=H, F, Cl, Br, I). J. Chem. Phys..

[86] Sanchez M., Provasi P.F., Aucar G.A., Sauer S.P.A. (2005). On the Usage of Locally Dense Basis Sets in the Calculation of NMR Indirect Nuclear Spin–Spin Coupling Constants: Vicinal Fluorine–Fluorine Couplings. Adv. Quantum Chem..

[87] Pierens G.K. (2014). ^1^H and ^13^C NMR scaling factors for the calculation of chemical shifts in commonly used solvents using density functional theory. J. Comput. Chem..

[88] Konstantinov I.A., Broadbelt L.J. (2011). Regression formulas for density functional theory calculated ^1^H and ^13^C NMR chemical shifts in toluene-d_8_. J. Phys. Chem. A.

[89] Paschoal D., Guerra C.F., de Oliveira M.A.L., Ramalho T.C., Dos Santos H.F. (2016). Predicting Pt-195 NMR Chemical Shift Using New Relativistic All-electron Basis Set. J. Comput. Chem..

[90] Colebatch A.L., Cade I.A., Hill A.F., Bhadbhade M.M. (2013). *η*^2^-Allenyl- and *η*^2^-Alkynylphosphonium Complexes of Platinum. Organometallics.

[91] Seidl A., Görling A., Vogl P., Majewski J.A., Levy M. (1996). Generalized Kohn-Sham Schemes and the Band-Gap Problem. Phys. Rev. B.

[92] Frisch M.J., Trucks G.W., Schlegel H.B., Scuseria G.E., Robb M.A., Cheeseman J.R., Scalmani G., Barone V., Petersson G.A., Nakatsuji H. (2016). Gaussian.

[93] Adamo C., Barone V. (1999). Toward reliable density functional methods without adjustable parameters: The PBE0 model. J. Chem. Phys..

[94] Hehre W.J., Ditchfield R., Pople J.A. (1972). Self-Consistent Molecular Orbital Methods. XII. Further Extensions of Gaussian-Type Basis Sets for Use in Molecular Orbital Studies of Organic Molecules. J. Chem. Phys..

[95] Clark T., Chandrasekhar J., Spitznagel G.W., Schleyer P.V.R. (1983). Efficient diffuse function-augmented basis sets for anion calculations. III. The 3-21+G basis set for first-row elements, Li–F. J. Comput. Chem..

[96] Francl M.M., Pietro W.J., Hehre W.J., Binkley J.S., Gordon M.S., DeFrees D.J., Pople J.A. (1982). Self-consistent molecular orbital methods. XXIII. A polarization-type basis set for second-row elements. J. Chem. Phys..

[97] Frisch M.J., Pople J.A., Binkley J.S. (1984). Self-consistent molecular orbital methods 25. Supplementary functions for Gaussian basis sets. J. Chem. Phys..

[98] Krishnan R., Binkley J.S., Seeger R., Pople J.A. (1980). Self-consistent molecular orbital methods. XX. A basis set for correlated wave functions. J. Chem. Phys..

[99] McLean A.D., Chandler G.S. (1980). Contracted Gaussian basis sets for molecular calculations. I. Second row atoms, Z = 11–18. J. Chem. Phys..

[100] Spitznagel G.W., Clark T., von Raguй Schleyer P., Hehre W.J. (1987). An evaluation of the performance of diffuse function-augmented basis sets for second row elements, Na-Cl. J. Comput. Chem..

[101] Ditchfield R., Hehre W.J., Pople J.A. (1971). Self-Consistent Molecular-Orbital Methods. IX. An Extended Gausian-Type Basis for Molecular-Orbital Studies of Organic Molecules. J. Chem. Phys..

[102] Andrae D., Haeussermann U., Dolg M., Stoll H., Preuss H. (1990). Energy-adjusted ab initio pseudopotentials for the second and third row transition elements. Theor. Chem. Acc..

[103] Miertuš S., Scrocco E., Tomasi J. (1981). Electrostatic interaction of a solute with a continuum. A direct utilization of AB initio molecular potentials for the prevision of solvent effects. Chem. Phys..

[104] Hansen A.E., Bouman T.D. (1985). Localized orbital/local origin method for calculation and analysis of NMR shieldings. Applications to ^13^C shielding tensors. J. Chem. Phys..

[105] Repisky M., Komorovsky S., Malkin V.G., Malkina O.L., Kaupp M., Ruud K., Bast R., Ekstrom U., Kadek M., Knecht S. (2019). MAG-ReSpect.

[106] Dyall K.G. (2004). Relativistic Double-Zeta, Triple-Zeta, and Quadruple-Zeta Basis Sets for the 5d Elements Hf-Hg. Theor. Chem. Acc..

[107] Dunning T.H. (1989). Gaussian basis sets for use in correlated molecular calculations. I. The atoms boron through neon and hydrogen. J. Chem. Phys..

[108] Woon D.E., Dunning T.H. (1993). Gaussian basis sets for use in correlated molecular calculations. III. The atoms aluminum through argon. J. Chem. Phys..

[109] Wilson A.K., Woon D.E., Peterson K.A., Dunning T.H. (1999). Gaussian Basis Sets for Use in Correlated Molecular Calculations. IX. The Atoms Gallium through Krypton. J. Chem. Phys..

[110] Jensen F. (2001). Polarization Consistent Basis Sets: Principles. J. Chem. Phys..

[111] Jensen F. (2002). Polarization Consistent Basis Sets. II. Estimating the Kohn–Sham Basis Set Limit. J. Chem. Phys..

[112] Jensen F., Helgaker T. (2004). Polarization Consistent Basis Sets. V. The Elements Si–Cl. J. Chem. Phys..

[113] Jensen F. (2012). Polarization Consistent Basis Sets. VII. The Elements K, Ca, Ga, Ge, As, Se, Br, and Kr. J. Chem. Phys..

[114] Pritchard B.P., Altarawy D., Didier B., Gibson T.D., Windus T.L. (2019). A New Basis Set Exchange: An Open, Up-to-date Resource for the Molecular Sciences Community. J. Chem. Inf. Model..

[115] Feller D. (1996). The Role of Databases in Support of Computational Chemistry Calculations. J. Comput. Chem..

[116] Schuchardt K.L., Didier B.T., Elsethagen T., Sun L., Gurumoorthi V., Chase J., Li J., Windus T.L. (2007). Basis Set Exchange:  A Community Database for Computational Sciences. J. Chem. Inf. Model..

[117] Wicht D.K., Paisner S.N., Lew B.M., Glueck D.S., Yap G.P.A., Liable-Sands L.M., Rheingold A.L., Haar C.M., Nolan S.P. (1998). Terminal Platinum(II) Phosphido Complexes:  Synthesis, Structure, and Thermochemistry. Organometallics.

[118] Song D., Wang S. (2003). Benzene C−H Activation by Two Isomeric Platinum(II) Complexes of Bis(N-7-Azaindolyl)Methane. Organometallics.

[119] Clark H.C., Ward J.E.H. (1974). ^13^C Nuclear Magnetic Resonance Studies of Organometallic Compounds. III. Cis-Methylplatinum(II) Derivatives. Can. J. Chem..

[120] Suslick B.A., Liberman-Martin A.L., Wambach T.C., Tilley T.D. (2017). Olefin Hydroarylation Catalyzed by (Pyridyl-Indolate)Pt(II) Complexes: Catalytic Efficiencies and Mechanistic Aspects. ACS Catal..

[121] Mukhopadhyay S., Lasri J., Guedes da Silva M.F.C., Januário Charmier M.A., Pombeiro A.J.L. (2008). Activation of C–CN Bond of Propionitrile: An Alternative Route to the Syntheses of 5-Substituted-1H-Tetrazoles and Dicyano-Platinum(II) Species. Polyhedron.

[122] Fujita M., Kim W.H., Sakanishi Y., Fujiwara K., Hirayama S., Okuyama T., Ohki Y., Tatsumi K., Yoshioka Y. (2004). Elimination−Addition Mechanism for Nucleophilic Substitution Reaction of Cyclohexenyl Iodonium Salts and Regioselectivity of Nucleophilic Addition to the Cyclohexyne Intermediate. J. Am. Chem. Soc..

[123] Riera X., López C., Caubet A., Moreno V., Solans X., Font-Bardia M. (2001). Platinum(II) and Palladium(II) Compounds Containing Chiral Thioimines. Eur. J. Inorg. Chem..

[124] Fuertes S., Chueca A.J., Sicilia V. (2015). Exploring the Transphobia Effect on Heteroleptic NHC Cycloplatinated Complexes. Inorg. Chem..

[125] Pazderski L., Pawlak T., Sitkowski J., Kozerski L., Szłyk E. (2009). ^1^H, ^13^C, ^15^N and ^195^Pt NMR Studies of Au(III) and Pt(II) Chloride Organometallics with 2-phenylpyridine. Magn. Reson. Chem..

[126] Carroll J., Gagnier J.P., Garner A.W., Moots J.G., Pike R.D., Li Y., Huo S. (2013). Reaction of N-Isopropyl-N-Phenyl-2,2′-Bipyridin-6-Amine with K_2_PtCl_4_: Selective C–H Bond Activation, C–N Bond Cleavage, and Selective Acylation. Organometallics.

[127] Hoogervorst W.J., Elsevier C.J., Lutz M., Spek A.L. (2001). New Cis- and Trans-Arylplatinum(II) Acetylide Compounds Containing a Bis(Imino)Aryl [NCN] Ligand. Organometallics.

[128] Zhang X., Wright A.M., DeYonker N.J., Hollis T.K., Hammer N.I., Webster C.E., Valente E.J. (2012). Synthesis, Air Stability, Photobleaching, and DFT Modeling of Blue Light Emitting Platinum CCC-N-Heterocyclic Carbene Pincer Complexes. Organometallics.

[129] Jia Y.-X., Yang X.-Y., Tay W.S., Li Y., Pullarkat S.A., Xu K., Hirao H., Leung P.-H. (2016). Computational and Carbon-13 NMR Studies of Pt–C Bonds in P–C–P Pincer Complexes. Dalton Trans..

[130] Brendel M., Engelke R., Desai V.G., Rominger F., Hofmann P. (2015). Synthesis and Reactivity of Platinum(II) Cis-Dialkyl, Cis-Alkyl Chloro, and Cis-Alkyl Hydrido Bis-N-Heterocyclic Carbene Chelate Complexes. Organometallics.

[131] Weiss D.T., Altmann P.J., Haslinger S., Jandl C., Pöthig A., Cokoja M., Kühn F.E. (2015). Structural Diversity of Late Transition Metal Complexes with Flexible Tetra-NHC Ligands. Dalton Trans..

[132] Lu T., Liu Z., Steren C.A., Fei F., Cook T.M., Chen X.-T., Xue Z.-L. (2018). Synthesis, Structural Characterization and NMR Studies of Group 10 Metal Complexes with Macrocyclic Amine N-Heterocyclic Carbene Ligands. Dalton Trans..

[133] Seyboldt A., Wucher B., Hohnstein S., Eichele K., Rominger F., Törnroos K.W., Kunz D. (2015). Evidence for the Formation of Anionic Zerovalent Group 10 Complexes as Highly Reactive Intermediates. Organometallics.

[134] Dahm G., Bailly C., Karmazin L., Bellemin-Laponnaz S. (2015). Synthesis, Structural Characterization and in Vitro Anti-Cancer Activity of Functionalized N-Heterocyclic Carbene Platinum and Palladium Complexes. J. Organomet. Chem..

[135] Muenzner J.K., Rehm T., Biersack B., Casini A., de Graaf I.A.M., Worawutputtapong P., Noor A., Kempe R., Brabec V., Kasparkova J. (2015). Adjusting the DNA Interaction and Anticancer Activity of Pt(II) N-Heterocyclic Carbene Complexes by Steric Shielding of the Trans Leaving Group. J. Med. Chem..

[136] Bouché M., Dahm G., Wantz M., Fournel S., Achard T., Bellemin-Laponnaz S. (2016). Platinum(IV) N-Heterocyclic Carbene Complexes: Their Synthesis, Characterisation and Cytotoxic Activity. Dalton Trans..

[137] Crumpton-Bregel D.M., Goldberg K.I. (2003). Mechanisms of C−C and C−H Alkane Reductive Eliminations from Octahedral Pt(IV):  Reaction via Five-Coordinate Intermediates or Direct Elimination?. J. Am. Chem. Soc..

[138] Scheuermann M.L., Luedtke A.T., Hanson S.K., Fekl U., Kaminsky W., Goldberg K.I. (2013). Reactions of Five-Coordinate Platinum(IV) Complexes with Molecular Oxygen. Organometallics.

[139] Green M., Howard J.A.K., Mitrprachachon P., Pfeffer M., Spencer J.L., Stone F.G.A., Woodward P. (1979). Organo-Complexes of Platinum Derived from Methyl Vinyl Ketone and Bis(Cyclo-Octa-1,5-Diene)Platinum; X-Ray Crystal Structure of (1,3-Diacetylbutane-1,4-Diyl)Bis(Triphenylphosphine)Platinum. J. Chem. Soc. Dalton Trans..

[140] Ogoshi S., Morita M., Kurosawa H. (2003). Synthesis, Structure, and Reactivity of a η^3^-1-Hydroxyallyl Complex:  Protonation of an α,β-Unsaturated Carbonyl Compound Bound to Palladium(0) and Platinum(0). J. Am. Chem. Soc..

