# Supplementary material for: DFT Approach for Predicting 13C NMR Shifts of Atoms Directly Coordinated to Pt: Scopes and Limitations
Source: Molecules. 2024 Dec 23;29(24):6052. doi: 10.3390/molecules29246052 (PMC11678254; doi:10.3390/molecules29246052)
Supplement: Supplementary file 1 [file molecules-29-06052-s001.zip › SI.pdf]

Supplementary Information for:

DFT approach for predicting ^{13}C NMR shifts of atoms directly coordinated to Pt: Scopes and limitations

*Svetlana A. Kondrashova, Shamil K. Latypov**

Arbuzov Institute of Organic and Physical Chemistry, FRC Kazan Scientific Center of RAS,
Kazan, Tatarstan 420088, Russian Federation

Table of contents **pages**

Calculational details.......... 2

Figure S1. Model Pt complexes (Mes = 1,3,5-trimethylbenzene, Mes* = 1,3,5-tri-*tert*-butylbenzene, Dipp = 2,6-diisopropylphenyl, Xyl = 2,6-dimethylphenyl, Np=CH_2-^{t}Bu, Bn = Benzyl). 3

Figure S1 (continued).......... 4

Table S1. Experimental, calculated ^{13}C NMR shifts (ppm) for all model Pt complexes **1-80**. 5

Table S2. Experimental and calculated ^{13}C NMR shifts (ppm) for all carbon atoms in complexes **10, 11**, **39, 59** and **67** (carbon atoms directly bonded to Pt are in **bold**).......... 9

Table S3. Experimental and calculated (using three different levels) ^{13}C NMR shifts (ppm) for complexes **19, 20, 57** and **68**. 12

Table S4. Empirical scaling factors obtained by the linear regression analysis of calculated and experimental $\delta^{13}C$ NMR shifts for all model complexes. 12

Table S5. Experimental and calculated ^{13}C NMR shifts (ppm) for "training" set of Pt complexes (**1-3**, **8**, **19-20**, **28**, **31**, **35**, **38-39**, **53**, **59-61**, **64-65**, **68**, **75**, **77-80**). 13

References.......... 15

Calculational details

Non relativistic quantum chemical calculations were carried out within the framework of the generalized Kohn Sham (KS) density functional theory [1], with the Gaussian 16 [2] (Revision A.03) software packages by using PBE0 [3] functional and Pople's basis sets [4-11]. For the Pt center, the quasi-relativistic Stuttgart–Dresden ECP60MWB was used with corresponding (8s7p6d)/(6s5p3d) GTO valence basis set [12] (denoted as "SDD") and NMR-DKH (TZ2P) basis set [13]. Wherever possible, geometry optimization was started from an X-ray structure. For most of the complexes, the calculations were carried out for all possible conformers/isomers, and results for the lowest energy forms were used in the analysis. To take into account the medium effects, calculations were carried out in the framework of the Polarizable Continuum Model [14] (denoted as "PCM") with the same solvent as that used in NMR experiments. ^{13}C NMR shifts were calculated by the GIAO method [15]. All ^{13}C data were referenced to tetramethylsilane (TMS), which was calculated under the same conditions.

Fully relativistic DFT ^{13}C NMR shifts calculations have been carried out at the matrix Dirac-Kohn-Sham (mDKS) level [16] with the ReSpect-MAG code [17]. The four-component mDKS calculations have been done with PBE0 functional. The uncontracted Dyall valence double-ζ basis set [18] was used for the Pt center. For ligand atoms two locally dense basis sets (LDBS) schemes [19-23] were used: 1) the Dunning's triple-ζ quality basis sets (ucc-pVTZ) on spectator atoms and atoms vicinal to Pt center and double-ζ quality basis sets (ucc-pVDZ) [24-26] on the remaining atoms were applied (denoted as "TZ_DZ"); 2) the Dunning's triple-ζ quality basis sets (ucc-pVTZ) on spectator atoms and atoms vicinal to Pt center, double-ζ quality basis sets (ucc-pVDZ) on the next layer and unpolarized Jensen basis set (upc-0) [27-30] on the remaining atoms were applied (denoted as "TZ_DZ_UPC"). For relativistic shielding calculations, the PBE0/{6-31+G(d); Pt(SDD)} geometry were used unless otherwise stated.

The NMR-DKH (TZ2P) basis sets were downloaded from the EMSL basis set library for the Gaussian package [31-33].

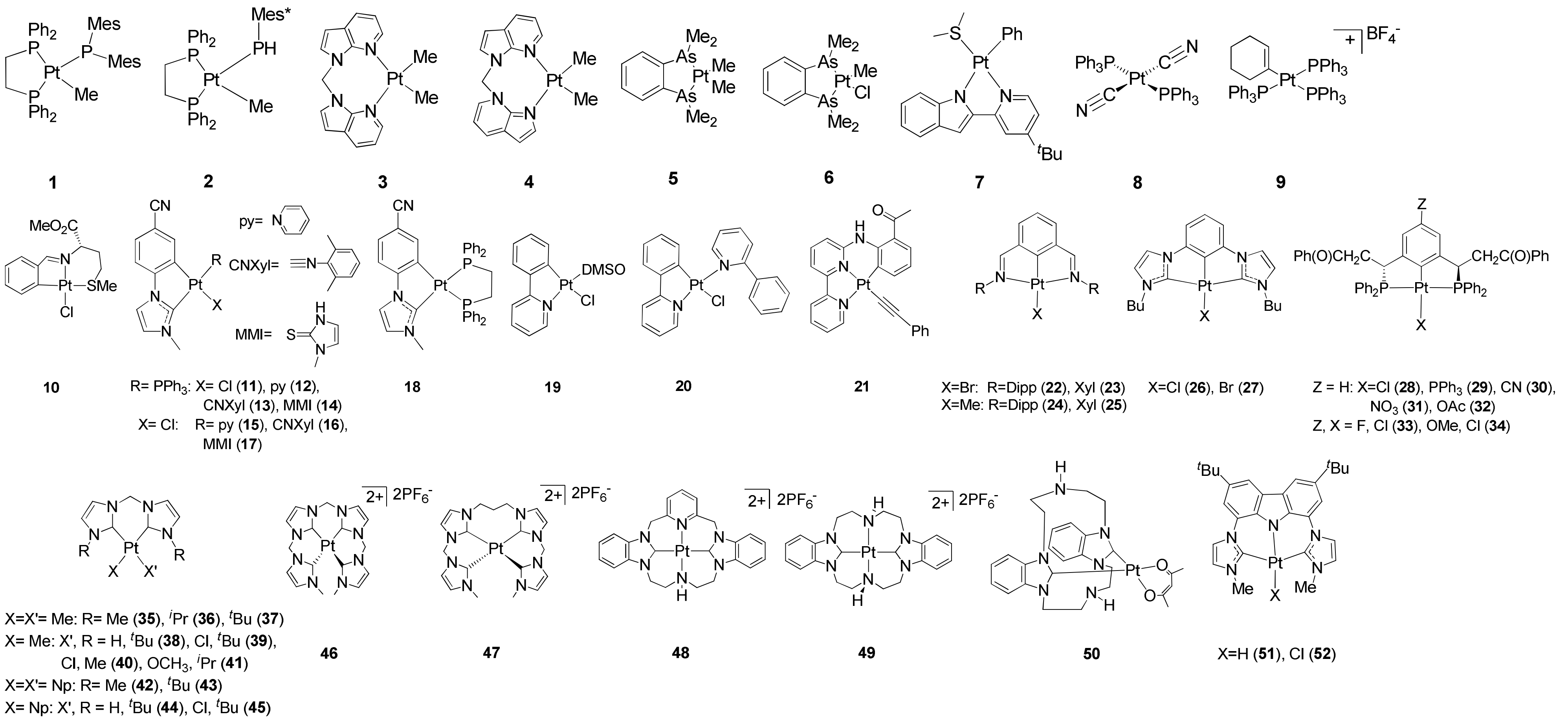

Figure S1. Model Pt complexes (Mes = 1,3,5-trimethylbenzene, Mes* = 1,3,5-tri-*tert*-butylbenzene, Dipp = 2,6-diisopropylphenyl, Xyl = 2,6-dimethylphenyl, Np=CH_2-^{t}Bu, Bn = Benzyl).

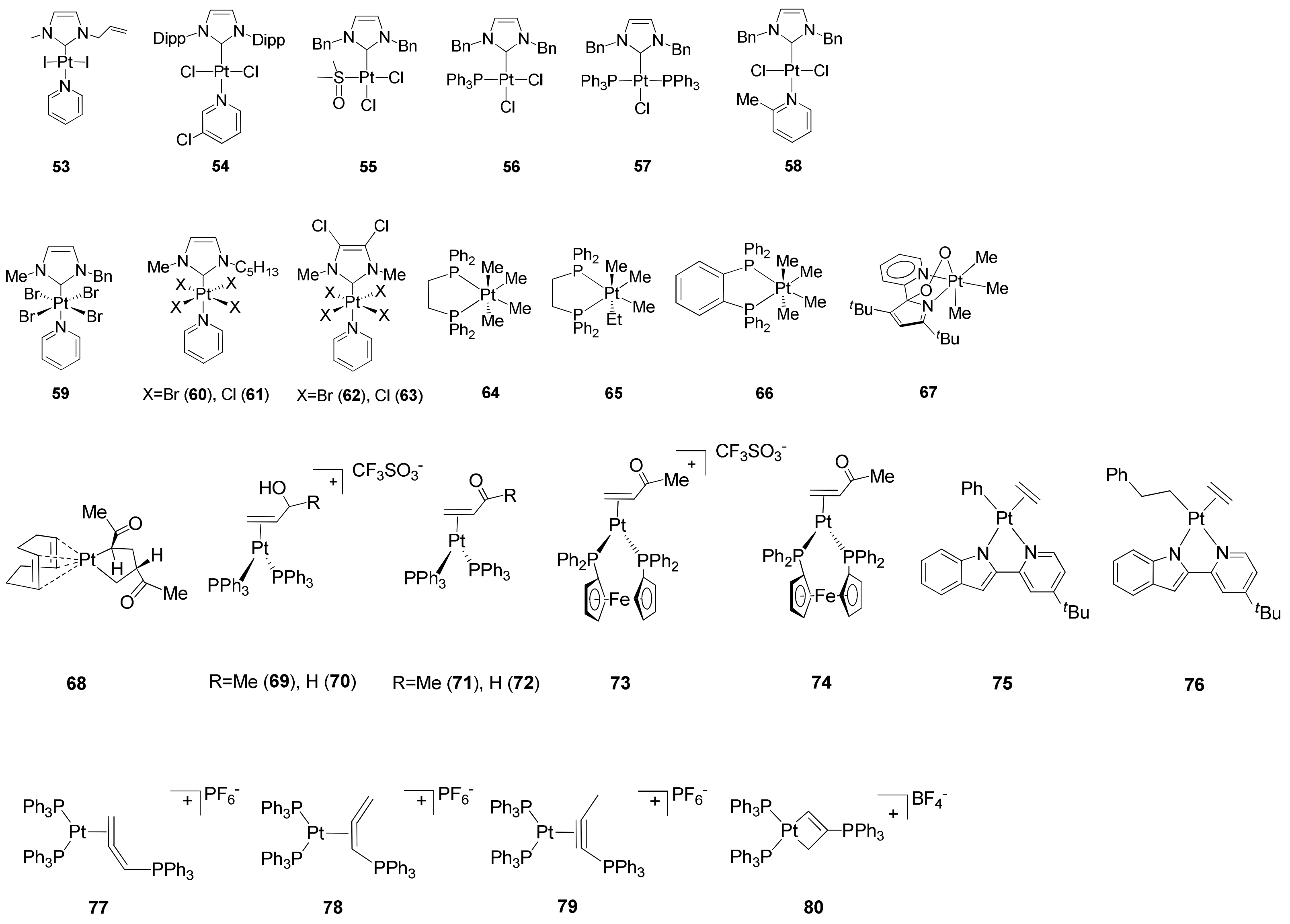

Figure S1 (continued).

Table S1. Experimental, calculated ^{13}C NMR shifts (ppm) for all model Pt complexes **1-80**.

Complex	Atom	Experimental	Calculated						Reference
			**KS**[a] level	***KS***[a] *level, scaled*	$\delta^{KS (scal.)} - \delta^{exp.}$	**mDKS/** TZ_DZ	***mDKS/*** *TZ_DZ, scaled*	$\delta^{mDKS (scal).} - \delta^{exp.}$	
**1**	Me	-0.3	10.9	*-6.2*	**-5.9**	-2.3[b]	*-3.0*	**-2.7**	34
**2**	Me	0.8	11.9	*-5.2*	**-6.0**	0.9[b]	*0.0*	**-0.8**	34
**3**	Me	-18.8	11.1	*-6.0*	**12.8**	-18.9	*-18.7*	**0.1**	35
**4**	Me	-17.6	10.8	*-6.3*	**11.4**	-19.3	*-19.1*	**-1.4**	35
**5**	Me	-8.8	8.8	*-8.2*	**0.6**	-4.5	*-5.1*	**3.7**	36
**6**	Me	-5.4	11.4	*-5.7*	**-0.3**	-1.1	*-1.9*	**3.5**	36
**7**	Ph	149.9	182.3	*158.7*	**8.8**	156.2	*146.5*	**-3.4**	37
**8**	C≡N	125.7	148.5	*126.2*	**0.5**	132.7	*124.3*	**-1.4**	38
**9**	C_6H_9	136.0	171.6	*148.4*	**12.4**	154.7	*145.1*	**9.1**	39
**10**	Ar-Pt-S	152.1	184.9	*161.2*	**9.1**	162.7	*152.6*	**0.5**	40
**11**	NHC	170.1	186.1	*162.3*	**-7.8**	178.0	*167.1*	**-3.0**	41
	Ar	128.5	163.4	*140.5*	**12.0**	138.8	*130.1*	**1.6**	
**12**	NHC	171.2	183.1	*159.4*	**-11.8**	177.5	*166.6*	**-4.6**	41
**13**	NHC	169.3	182.6	*158.9*	**-10.4**	175.5	*164.7*	**-4.6**	41
	Ar	138.6	156.1	*133.5*	**-5.1**	146.7	*137.5*	**-1.1**	
**14**	NHC	170.7	182.9	*159.2*	**-11.5**	175.6	*164.8*	**-5.9**	41
**15**	NHC	152.7	190.7	*166.7*	**14.0**	161.9	*151.9*	**-0.8**	41
	Ar	131.3	166.6	*143.6*	**12.3**	142.8	*133.9*	**2.6**	
**16**	NHC	167.7	185.3	*161.5*	**-6.2**	178.9	*167.9*	**0.2**	41
	Ar	128.7	163.3	*140.4*	**11.7**	139.8	*131.0*	**2.3**	
**17**	NHC	159.3	186.5	*162.7*	**3.4**	165.4	*155.2*	**-4.1**	41
	Ar	126.7	164.4	*141.4*	**14.7**	134.9	*126.4*	**-0.3**	
**18**	NHC	172.7	184.1	*160.4*	**-12.3**	178.8	*167.8*	**-4.9**	41
	Ar	143.9	159.1	*136.3*	**-7.6**	150.0	*140.7*	**-3.2**	
**19**	Ar-2ppy	140.2	176.3	*152.9*	**12.7**	150.6	*141.2*	**1.0**	42
**20**	Ar-2ppy	140.6	180.9	*157.3*	**16.7**	154.4	*144.8*	**4.2**	43
**21**	Ar	135.1	159.2	*136.4*	**1.3**	129.6	*121.4*	**-13.7**	44
	-C≡C-Ph	118.5	145.5	*123.3*	**4.8**	117.0	*109.5*	**-9.0**	
**22**	Ar-Pt-Br	179.0	206.1	*181.5*	**2.5**	192.8	*181.0*	**2.0**	45
**23**	Ar-Pt-Br	178.4	205.8	*181.3*	**2.8**	193.0	*181.2*	**2.8**	45

**24**	*Ar*-Pt-Me	204.6	213.5	*188.7*	**-15.9**	221.2	*207.8*	**3.2**	45
	Me	10.2	3.8	*-13.0*	**-23.2**	11.7	*10.2*	**0.0**	
**25**	*Ar*-Pt-Me	204.0	213.4	*188.6*	**-15.4**	221.6	*208.2*	**4.2**	45
	Me	8.7	1.9	*-14.8*	**-23.5**	9.5	*8.1*	**-0.6**	
**26**	NHC	171.7	187.6	*163.8*	**-8.0**	183.4	*172.2*	**0.5**	46
	Ar	133.8	165.3	*142.3*	**8.5**	147.1	*137.9*	**4.1**	
**27**	NHC	170.5	187.4	*163.6*	**-6.9**	182.0	*170.8*	**0.3**	46
	Ar	134.0	163.9	*141.0*	**7.0**	148.2	*139.0*	**5.0**	
**28**	Ar-Pt-Cl	145.9	176.0	*152.6*	**6.7**	155.1[b]	*145.5*	**-0.4**	47
**29**	Ar-Pt-PPh_3	157.8	174.4	*151.1*	**-6.7**	164.6[b]	*154.4*	**-3.4**	47
**30**	Ar-Pt-CN	159.5	177.3	*153.8*	**-5.7**	169.7[b]	*159.2*	**-0.3**	47
**31**	Ar-Pt-NO_3	135.3	173.8	*150.5*	**15.2**	143.6[b]	*134.6*	**-0.7**	47
**32**	Ar-Pt-OAc	139.0	174.3	*151.0*	**12.0**	147.0[b]	*137.8*	**-1.2**	47
**33**	Ar-Pt-Cl	140.7	169.4	*146.3*	**5.6**	150.0[b]	*140.7*	**0.0**	47
**34**	Ar-Pt-Cl	136.6	164.8	*141.8*	**5.2**	144.7[b]	*135.7*	**-0.9**	47
**35**	NHC	185.08	200.9	*176.5*	**-8.5**	198.0	*185.9*	**0.9**	48
	Me	-8.34	6.9	*-10.0*	**-1.7**	-7.9	*-8.3*	**0.0**	
**36**	NHC	180.52	199.7	*175.4*	**-5.1**	196.6	*184.6*	**4.1**	48
	Me	-7.51	6.1	*-10.8*	**-3.3**	-8.8	*-9.2*	**-1.6**	
**37**	NHC	186.89	203.4	*178.9*	**-7.9**	199.8	*187.6*	**0.8**	48
	Me	-8.25	10.7	*-6.3*	**1.9**	-7.2	*-7.6*	**0.6**	
**38**	NHC (*trans* H)	189.66	206.1	*181.5*	**-8.1**	203.0	*190.7*	**1.0**	48
	NHC (*trans* Me)	183.18	199.2	*174.9*	**-8.3**	194.7	*182.8*	**-0.3**	
	Me	-23.91	-3.6	*-20.1*	**3.8**	-24.8	*-24.2*	**-0.3**	
**39**	NHC (*trans* Me)	182.61	194.8	*170.7*	**-11.9**	196.5	*184.5*	**1.9**	48
	NHC (*trans* Cl)	155.79	198.0	*173.8*	**18.0**	172.2	*161.6*	**5.8**	
	Me	-6.74	10.0	*-7.0*	**-0.3**	-6.8	*-7.3*	**-0.5**	
**40**	NHC (*trans* Me)	181.77	193.8	*169.7*	**-12.1**	196.4	*184.4*	**2.7**	48
	NHC (*trans* Cl)	155.21	196.1	*171.9*	**16.7**	172	*161.4*	**6.2**	
	Me	-6.07	9.3	*-7.7*	**-1.6**	-3.9	*-4.5*	**1.5**	
**41**	NHC (*trans* Me)	181.86	190.9	*166.9*	**-14.9**	192.7	*180.9*	**-0.9**	48
	NHC (*trans* OCH_3)	156.28	197.4	*173.2*	**16.9**	170.4	*159.9*	**3.6**	
	Me	-5.27	8.1	*-8.8*	**-3.6**	-4.7	*-5.3*	**0.0**	
**42**	NHC	187.88	204.5	*180.0*	**-7.9**	200.1	*187.9*	**0.0**	48
	CH_2	34.82	50.4	*31.8*	**-3.0**	39.2	*36.1*	**1.3**	
**43**	NHC	188.44	205.8	*181.3*	**-7.2**	200.1	*187.9*	**-0.5**	48

	CH_2	34.18	50.3	*31.7*	**-2.4**	35.8	*32.9*	**-1.3**	
**44**	NHC (*trans* CH_2)	184.18	199.7	*175.4*	**-8.8**	194.7	*182.8*	**-1.3**	48
	NHC (*trans* H)	191.14	204.1	*179.6*	**-11.5**	201.7	*189.4*	**-1.7**	
	CH_2	30.45	39.6	*21.4*	**-9.0**	23	*20.8*	**-9.6**	
**45**	NHC (*trans* CH_2)	183.73	195.2	*171.1*	**-12.7**	196.3	*184.3*	**0.6**	48
	NHC (*trans* Cl)	157.48	197.5	*173.3*	**15.8**	171.5	*160.9*	**3.5**	
	CH_2	32.54	48.7	*30.2*	**-2.3**	34.0	*31.2*	**-1.3**	
**46**	NHC	161.20	180.0	*156.4*	**-4.8**	167.2	*156.9*	**-4.3**	49
	NHC	155.78	179.4	*155.9*	**0.1**	164.3	*154.2*	**-1.6**	
**47**	NHC	163.65	181.2	*157.6*	**-6.1**	169.1	*158.7*	**-5.0**	49
	NHC	162.23	180.6	*157.0*	**-5.2**	167.6	*157.3*	**-5.0**	
**48**	NHC	170.48	181.3	*157.7*	**-12.8**	173.3	*162.6*	**-7.8**	50
**49**	NHC	160.30	174.4	*151.1*	**-9.2**	160.1	*150.2*	**-10.1**	50
**50**	NHC	153.40	197.3	*173.1*	**19.7**	162.6	*152.5*	**-0.9**	50
**51**	NHC	170.80	191.0	*167.0*	**-3.8**	177.5	*166.6*	**-4.2**	51
**52**	NHC	159.90	185.7	*161.9*	**2.0**	170.4	*159.9*	**0.0**	51
**53**	NHC	136.0	186.7	*162.9*	**26.9**	145.6	*136.5*	**0.5**	52
**54**	NHC	150.5	192.2	*168.2*	**17.7**	153.6	*144.1*	**-6.4**	53
**55**	NHC	144.9	184.2	*160.5*	**15.6**	158.7	*148.9*	**4.0**	53
**56**	NHC	149.9	186.4	*162.6*	**12.7**	162.4	*152.4*	**2.5**	53
**57**	NHC	145.0	178.2	*154.7*	**9.7**	154.6	*145.0*	**0.0**	53
**58**	NHC	151.4	189.4	*165.5*	**14.1**	153.4	*143.9*	**-7.5**	53
**59**	Pt(IV)-NHC	109.3	170.5	*147.3*	**38.0**	124.3	*116.4*	**7.1**	54
**60**	Pt(IV)-NHC	109.2	169.8	*146.6*	**37.4**	124.0	*116.1*	**6.9**	54
**61**	Pt(IV)-NHC	111.5	170.4	*147.2*	**35.7**	124.4	*116.5*	**5.0**	54
**62**	Pt(IV)-NHC	120.7	171.1	*147.9*	**27.2**	124.9	*117.0*	**-3.7**	54
**63**	Pt(IV)-NHC	112.9	171.6	*148.4*	**35.5**	125.7	*117.7*	**4.8**	54
**64**	Pt(IV)-Me^{ax}	-4.6	13.5	*-3.7*	**0.9**	-4.7	*-5.3*	**-0.7**	55
	Pt(IV)-Me^{eq}	3.4	26.1	*8.5*	**5.1**	4.9	*3.8*	**0.4**	
**65**	Pt(IV)-Me^{ax}	-2.1	17.9	*0.6*	**2.7**	1.1	*0.2*	**2.3**	55
	Pt(IV)-Me^{eq}	4.0	26.7	*9.0*	**5.0**	7.6	*6.3*	**2.3**	
**66**	Pt(IV)-Me^{ax}	-4.5	11.9	*-5.2*	**-0.7**	-6.3	*-6.8*	**-2.3**	55
	Pt(IV)-Me^{eq}	3.4	26.6	*8.9*	**5.5**	5.9	*4.7*	**1.3**	
	Pt(IV)-Et^{ax}	-2.8	16.7	*-0.6*	**2.2**	-3.8	*-4.4*	**-1.6**	
**67**	Pt(IV)-Me (*trans* NHC)	-6.5	25.9	*8.3*	**14.8**	-3.7	*-4.3*	**2.2**	56
	Pt(IV)-Me (*trans* py)	-9.8	21.5	*4.0*	**13.8**	-8.4	*-8.8*	**1.0**	

	Pt(IV)-Me (*trans* O_2)	-15.2	15.0	*-2.2*	**13.0**	-13.2	*-13.3*	**1.9**	
**68**	C=C (*cis* CH)	104.8	107.0	*86.3*	**-18.6**	107.7	*100.8*	**-4.0**	57
	C=C (*cis* CH_2)	95.7	103.3	*82.7*	**-13.0**	102.5	*95.8*	**0.1**	
	C=C (*cis* CH_2)	94.9	98.1	*77.7*	**-17.2**	97.6	*91.2*	**-3.7**	
	CH	60.2	68.4	*49.1*	**-11.1**	65.0	*60.5*	**0.3**	
	CH_2	35.4	53.0	*34.3*	**-1.1**	44.5	*41.1*	**5.7**	
**69**	CH	74.5	83.5	*63.7*	**-10.8**	83.2	*77.6*	**3.1**	58
	CH_2	41.1	47.2	*28.8*	**-12.4**	43.9	*40.6*	**-0.5**	
**70**	CH	83.3	89.6	*69.5*	**-13.8**	90.2	*84.2*	**0.9**	58
	CH_2	48.3	47.6	*29.1*	**-19.2**	45.4	*42.0*	**-6.3**	
**71**	CH	60.3	67.5	*48.3*	**-12.0**	65.7	*61.1*	**0.8**	58
	CH_2	38.4	47.6	*29.1*	**-9.3**	44.2	*40.8*	**2.4**	
**72**	CH	62.0	65.5	*46.3*	**-15.7**	63.1	*58.7*	**-3.3**	58
	CH_2	39.2	45.1	*26.7*	**-12.5**	41.6	*38.4*	**-0.8**	
**73**	CH	75.8	83.4	*63.6*	**-12.2**	83.1	*77.5*	**1.7**	58
	CH_2	42.6	49.7	*31.2*	**-11.4**	47.2	*43.7*	**1.1**	
**74**	CH	59.2	58.5	*39.6*	**-19.6**	56.7	*52.6*	**-6.6**	58
	CH_2	36.2	43.5	*25.2*	**-11.0**	40.0	*36.9*	**0.7**	
**75**	Ph	139.1	171.0	*147.8*	**8.7**	140.8	*132.0*	**-7.1**	37
	H_2C=CH_2	63.9	73.3	*53.8*	**-10.1**	66.6	*62.0*	**-1.9**	
**76**	CH_2	7.5	40.7	*22.5*	**15.0**	13.7	*12.1*	**4.6**	37
	H_2C=CH_2	60.2	70.1	*50.8*	**-9.4**	61.9	*57.5*	**-2.7**	
**77**	=C=	197.9	220.8	*195.7*	**-2.2**	217.5[b]	*204.3*	**6.4**	59
	=CH_2	14.0	22.3	*4.8*	**-9.2**	16.5[b]	*14.7*	**0.7**	
**78**	=C=	158.4	174.2	*150.9*	**-7.5**	166.2[b]	*155.9*	**-2.5**	59
	=CH-	2.8	16.3	*-1.0*	**-3.8**	2.6[b]	*1.6*	**-1.2**	
**79**	≡C-Me	172.0	194.8	*170.7*	**-1.3**	188.2[b]	*176.7*	**4.7**	59
	≡C-PPh_3	91.7	104.6	*83.9*	**-7.8**	93.5[b]	*87.4*	**-4.3**	
**80**	-CH=	161.1	192.9	*168.8*	**7.7**	174.5[b]	*163.8*	**2.7**	59
	-CH_2-	-12.6	5.7	*-11.2*	**1.4**	-12.0[b]	*-12.2*	**0.4**	
RMSE			**25.5**	***12.6***		**8.9**	***3.7***		
R^2			**0.9697**	***0.9697***		**0.9973**	***0.9973***		

[a] PBE0/{6-311G(2d,2p); Pt(SDD)}//PBE0/{6-31+G(d); Pt(SDD)} combination;
[b] with the "TZ_DZ_UPC" LDBS.

Table S2. Experimental and calculated ^{13}C NMR shifts (ppm) for all carbon atoms in complexes **10, 11**, **39, 59** and **67** (carbon atoms directly bonded to Pt are in **bold**).

	Complex	Atom	Experimental	Calculated at **KS** level[a]
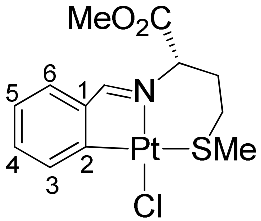 **10**	**10**	SMe	21.2	24.4
		SCH_2	27.7	34.0
		CH_2	31.4	30.8
		OMe	53.3	53.2
		CH	66.3	69.7
		C1	143.0	143.2
		**C2**	**152.1**	**184.9**
		C3	128.2	132.7
		C4	124.3	126.7
		C5	133.4	140.7
		C6	132.2	139.5
		CH=N	181.9	180.6
		CO_2	170.0	175.3
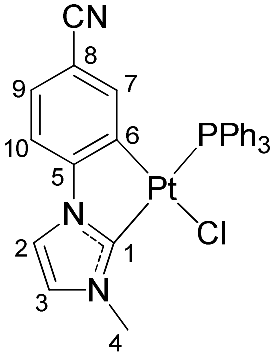 **11**	**11**	C4	38.6	42.7
		C8	108.0	115.5
		C10	111.0	113.5
		C2	113.9	113.6
		CN	118.8	123.6
		C3	124.3	126.5
		C9	127.8	131.4
		$C_m(PPh_3)$	128.1	132.5
		**C6**	**128.5**	**163.4**
		$C_i(PPh_3)$	130.1	137.8
		$C_p(PPh_3)$	130.7	135.4
		$C_o(PPh_3)$	135.5	141.7
		C7	141.0	149.6
		C5	150.5	151.6

		**C1**	**170.1**	**186.1**
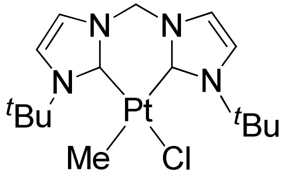 **39**	**39**	**Me**	**-6.74**	**10.0**
		N-C($\underline{C}H_3$)$_3$	31.52	32.3
		N-C($\underline{C}H_3$)$_3$	31.82	33.5
		N-$\underline{C}$(CH_3)$_3$	59.53	61.5
		CH_2	64.44	65.7
		CH-Im	118.37	118.7
		CH-Im	118.71	118.9
		CH-Im	118.87	120.7
		**NHC (*trans* Cl)**	**155.79**	**194.8**
		**NHC (*trans* Me)**	**182.61**	**198.0**
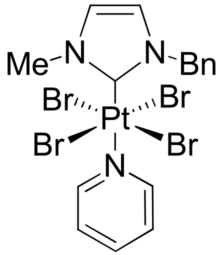 **59**	**59**	N-CH_3	44.1	45.1
		N-CH_2	59.1	62.3
		**C(NHC)-Pt**	**109.3**	**170.5**
		CH_{im}	124.4	128.1
		CH_{ar}	124.8	132.7
		CH_{im}	125.7	128.5
		CH_{ar}	128.7	133.2
		CH_{ar}	128.8	133.6
		C_{pyr}	136.7	126.9
		C_{pyr}	139.8	142.8
		C_{pyr}	154.3	160.5
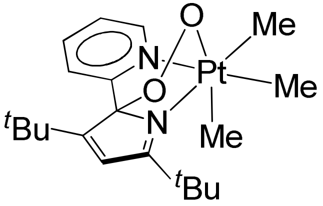 **67**	**67**	**Pt(IV)-Me (*trans* O_2)**	**-15.2**	**15.0**
		**Pt(IV)-Me (*trans* py)**	**-9.8**	**21.5**
		**Pt(IV)-Me (*trans* NHC)**	**-6.5**	**25.9**
		^{t}Bu	28.1	28.5
		^{t}Bu	32.6	32.8
		C-^{t}Bu	35.5	39.5
		C-^{t}Bu	36.6	39.9
		NC*sp*^{3}	119.1	115.0
		Cp-H	122.8	127.6
		Cp-H	125.3	127.6
		N-CC	127.0	131.5

		Cp-H	137.7	140.5
		Cp-H	145.5	150.3
		Cp	158.9	167.0
		CC-^{t}Bu	175.0	186.6
		CC-^{t}Bu	184.2	189.2

[a] PBE0/{6-311G(2d,2p); Pt(SDD)}//PBE0/{6-31+G(d); Pt(SDD)} combination.

Table S3. Experimental and calculated (using three different levels) ^{13}C NMR shifts (ppm) for complexes **19, 20, 57** and **68**.

Complex	Atom	Experimental	**KS**[a] level	**mDKS/** TZ_DZ	**mDKS/** TZ_DZ_UPC
**3**	Me	-18.8	11.1	-18.9	-18.9
**4**	Me	-17.6	10.8	-19.3	-19.3
**19**	Ar-2ppy	140.2	176.3	150.6	148.8
**68**	C=C (*cis* CH)	104.8	107.0	107.7	107.1
	C=C (*cis* CH_2)	95.7	103.3	102.5	102.5
	C=C (*cis* CH_2)	94.9	98.1	97.6	97.6
	CH	60.2	68.4	65.0	63.7
	CH_2	35.4	53.0	44.5	44.3

[a] PBE0/{6-311G(2d,2p); Pt(SDD)}//PBE0/{6-31+G(d); Pt(SDD)} level of theory.

Table S4. Empirical scaling factors obtained by the linear regression analysis of calculated and experimental δ^{13}C NMR shifts for all model complexes.

**Level of Theory**	R^2	**Slope**	**Intercept**	***RMSE***
**KS**	0.9697	1.04	-17.3	12.6
**mDKS**/TZ_DZ	0.9973	1.06	-0.9	3.7

Table S5. Experimental and calculated ^{13}C NMR shifts (ppm) for "training" set of Pt complexes (**1-3**, **8**, **19-20**, **28**, **31**, **35**, **38-39**, **53**, **59-61**, **64-65**, **68**, **75**, **77-80**).

Complex	Atom	Experimental	Calculated							Solvent
			geometry optimization							
			PBE0/ {**6-31+G(d);** Pt(SDD)}		PBE0/ {**6-311+G(2d)**; Pt(SDD)}		PBE0/ {6-31+G(d); Pt(SDD)}(**PCM**)		PBE0/ {**6-31+G(d);** Pt(SDD)}	
			shielding calculation							
			**KS**[a]	**mDKS/** TZ_DZ	**KS**[a]	**mDKS/** TZ_DZ	**KS**[a]	**mDKS/** TZ_DZ	**KS**[b] (Pt(NMR-DKH))	
**1**	Me	-0.3	10.9	-2.3[c]	10.9	-0.4[c]	10.4	0.5[c]	9.9	C_6H_6
**2**	Me	0.8	11.9	0.9[c]	11.6	2.5[c]	10.8	3.3[c]	11.7	C_6H_6
**3**	Me	-18.8	11.1	-18.9	10.9	-17.3	9.1	-16.6	7.4	THF
**8**	C≡N	125.7	148.5	132.7	147.3	132.6	150.2	131.7	151.3	$CHCl_3$
**19**	Ar-2ppy	140.2	176.3	150.6	175.8	152.2	175.2	151.7	184.4	$CHCl_3$
**20**	Ar-2ppy	140.6	180.9	154.4	180.5	155.9	179.6	155.0	188.9	$CHCl_3$
**28**	Ar-Pt-Cl	145.9	176.0	155.1[c]	175.5	156.3[c]	175.7	156.4[c]	183.1	$CHCl_3$
**31**	Ar-Pt-NO_3	135.3	173.8	143.6[c]	173.2	144.7[c]	173.0	144.8[c]	180.5	Acetone
**35**	NHC	185.1	200.9	198.0	200.0	198.4	196.1	199.4	207.2	THF
	Me	-8.3	6.9	-7.9	6.9	-6.2	4.8	-6.3	0.8	
**38**	NHC (*trans* H)	189.7	206.1	203.0	205.4	196.7	200.5	204.4	211.9	THF
	NHC (*trans* Me)	183.2	199.2	194.7	198.5	173.5	195.1	196.6	205.9	
	Me	-23.9	-3.6	-24.8	-3.3	-4.8	-5.7	-22.8	-12.9	
**39**	NHC (*trans* Me)	182.6	194.8	196.5	194	195.0	190.3	199.3	205.4	CH_2Cl_2
	NHC (*trans* Cl)	155.8	198.0	172.2	197.4	203.5	192.2	171.8	200.4	
	Me	-6.7	10.0	-6.8	10.2	-22.9	7.6	-5.4	8.2	
**53**	NHC	136.0	186.7	145.6	186.3	146.7	183.8	147.9	147.9	$CHCl_3$
**59**	Pt(IV)-NHC	109.3	170.5	124.3	169.6	123.3	165.2	125.9	170.7	CH_2Cl_2

**60**	Pt(IV)-NHC	109.2	169.8	124.0	169.5	124.0	164.7	125.2	170.1	$CHCl_3$
**61**	Pt(IV)-NHC	111.5	170.4	124.4	169.9	126.4	165.2	125.8	171	$CHCl_3$
**64**	Pt(IV)-Me^{ax}	-4.6	13.5	-4.7	13.8	-3.0	13.5	-2.4	14.6	C_6H_6
	Pt(IV)-Me^{eq}	3.4	26.1	4.9	25.2	5.8	24.1	7.0	26.6	
**65**	Pt(IV)-Me^{ax}	-2.1	17.9	1.1	18.4	2.6	17.2	3.1	19.1	THF
	Pt(IV)-Me^{eq}	4.4	26.7	7.6	26.8	9.1	25.2	9.8	27.6	
**68**	C=C (*cis* CH)	104.8	107.0	107.7	106.9	108.6	108.8	110.4	116.2	C_6H_6
	C=C (*cis* CH_2)	95.7	103.3	102.5	103.1	103.4	104.7	104.6	112.6	
	C=C (*cis* CH_2)	94.9	98.1	97.6	98.0	98.7	99.6	99.9	108.2	
	CH	60.2	68.4	65.0	68.5	66.1	67.6	67.3	71.9	
	CH_2	35.4	53.0	44.5	52.9	45.6	51.4	46.4	57.7	
**75**	Ph	7.5	40.7	13.7	40.9	15.2	40.3	15.8	44.0	C_6H_6
	$H_2C=CH_2$	60.2	70.1	61.9	70.7	64.2	71.2	64.5	81.5	
**77**	=C=	197.9	220.8	217.5[c]	218.9	217.2[c]	215.0	216.1[c]	229.8	$CHCl_3$
	$=CH_2$	14.0	22.3	16.5[c]	25.3	20.0[c]	23.6	20.2[c]	27.0	
**78**	=C=	158.4	174.2	166.2[c]	173.4	167.9[c]	177.1	169.4[c]	185.1	$CHCl_3$
	=CH-	2.8	16.3	2.6[c]	16.5	5.0[c]	17.0	6.3[c]	19.5	
**79**	≡C-Me	172.0	194.8	188.2[c]	195.7	190.9[c]	193.2	190.3[c]	193.1	$CHCl_3$
	$≡C-PPh_3$	91.7	104.6	93.5[c]	105.3	96.0[c]	105.9	97.0[c]	102.5	
**80**	-CH=	161.1	192.9	174.5[c]	192.2	175.2[c]	190.8	176.3[c]	199.5	$CHCl_3$
	$-CH_2-$	-12.6	5.7	-12.0[c]	5.4	-10.5[c]	5.8	-8.8[c]	-4.6	
R^2			**0.9651**	**0.9984**	**0.9654**	**0.9979**	**0.9688**	**0.9987**	**0.9723**	

[a] PBE0/{6-311G(2d,2p); Pt(SDD)}//PBE0/{6-31+G(d); Pt(SDD)} level of theory;
[b] PBE0/{6-311G(2d,2p);Pt(**NMR-DKH**)}//PBE0/{6-31+G(d);Pt(SDD)} level of theory;
[c] with the "TZ_DZ_UPC" LDBS.

References

(1) Seidl, A.; Görling, A.; Vogl, P.; Majewski, J. A.; Levy, M. Generalized Kohn-Sham Schemes and the Band-Gap Problem. *Phys. Rev. B* **1996**, *53*, 3764 – 3774.

(2) Frisch, M. J.; Trucks, G. W.; Schlegel, H. B.; Scuseria, G. E.; Robb, M. A.; Cheeseman, J. R.; Scalmani, G.; Barone, V.; Petersson, G. A.; Nakatsuji, H.; Li, X.; Caricato, M.; Marenich, A. V.; Bloino, J.; Janesko, B. G.; Gomperts, R.; Mennucci, B.; Hratchian, H. P.; Ortiz, J. V.; Izmaylov, A. F.; Sonnenberg, J. L.; Williams-Young, D.; Ding, F.; Lipparini, F.; Egidi, F.; Goings, J.; Peng, B.; Petrone, A.; Henderson, T.; Ranasinghe, D.; Zakrzewski, V. G.; Gao, J.; Rega, N.; Zheng, G.; Liang, W.; Hada, M.; Ehara, M.; Toyota, K.; Fukuda, R.; Hasegawa, J.; Ishida, M.; Nakajima, T.; Honda, Y.; Kitao, O.; Nakai, H.; Vreven, T.; Throssell, K.; Montgomery, J. A.; Peralta, J. E., Jr.; Ogliaro, F.; Bearpark, M. J.; Heyd, J. J.; Brothers, E. N.; Kudin, K. N.; Staroverov, V. N.; Keith, T. A.; Kobayashi, R.; Normand, J.; Raghavachari, K.; Rendell, A. P.; Burant, J. C.; Iyengar, S. S.; Tomasi, J.; Cossi, M.; Millam, J. M.; Klene, M.; Adamo, C.; Cammi, R.; Ochterski, J. W.; Martin, R. L.; Morokuma, K.; Farkas, O.; Foresman, J. B.; Fox, D. J. Gaussian 16, revision A.03; Gaussian, Inc.: Wallingford, CT.

(3) Adamo, C.; Barone, V. Toward reliable density functional methods without adjustable parameters: The PBE0 model. *J. Chem. Phys.* **1999**, *110*, 6158–6170.

(4) Hehre, W.J.; Ditchfield, R.; Pople, J.A. Self-Consistent Molecular Orbital Methods. XII. Further Extensions of Gaussian-Type Basis Sets for Use in Molecular Orbital Studies of Organic Molecules. *J. Chem. Phys.* **1972**, *56*, 2257–2261.

(5) Clark, T.; Chandrasekhar, J.; Spitznagel, G.W.; Schleyer, P.V.R. Efficient diffuse function-augmented basis sets for anion calculations. III. The 3-21+G basis set for first-row elements, Li–F. J. *Comput. Chem*. **1983**, *4*, 294–301.

(6) Francl, M.M.; Pietro, W.J.; Hehre, W.J.; Binkley, J.S.; Gordon, M.S.; DeFrees, D.J.; Pople, J.A. Self-consistent molecular orbital methods. XXIII. A polarization-type basis set for second-row elements. *J. Chem. Phys.* **1982**, *77*, 3654–3665.

(7) Frisch, M.J.; Pople, J.A.; Binkley, J.S. Self-consistent molecular orbital methods 25. Supplementary functions for Gaussian basis sets. *J. Chem. Phys.* **1984**, *80*, 3265–3269.

(8) Krishnan, R.; Binkley, J.S.; Seeger, R.; Pople, J.A. Self-consistent molecular orbital methods. XX. A basis set for correlated wave functions. *J. Chem. Phys.* **1980**, *72*, 650–654.

(9) McLean, A.D.; Chandler, G.S. Contracted Gaussian basis sets for molecular calculations. I. Second row atoms, Z = 11–18. *J. Chem. Phys.* **1980**, *72*, 5639–5648.

(10) Spitznagel, G.W.; Clark, T.; von Raguй Schleyer, P.; Hehre, W.J. An evaluation of the performance of diffuse function-augmented basis sets for second row elements, Na-Cl. *J. Comput. Chem.* **1987**, *8*, 1109–1116.

(11) Ditchfield, R.; Hehre, W.J.; Pople, J.A. Self-Consistent Molecular-Orbital Methods. IX. An Extended Gausian-Type Basis for Molecular-Orbital Studies of Organic Molecules. *J. Chem. Phys.* **1971**, *54*, 724–728.

(12) Andrae, D.; Haeussermann, U.; Dolg, M.; Stoll, H.; Preuss, H. Energy-adjusted *ab initio* pseudopotentials for the second and third row transition elements. *Theor. Chem. Acc.* **1990**, *77*, 123–141.

(13) Paschoal, D.; Guerra, C. F.; de Oliveira, M. A. L.; Ramalho, T. C.; Dos Santos, H. F. Predicting Pt-195 NMR Chemical Shift Using New Relativistic All-electron Basis Set. *J. Comp. Chem.* **2016**, *37*, 2360–2373.

(14) Miertuš, S.; Scrocco, E.; Tomasi, J. Electrostatic interaction of a solute with a continuum. A direct utilization of AB initio molecular potentials for the prevision of solvent effects. *Chem. Phys.* **1981**, *55*, 117−129.

(15) Hansen, A.E.; Bouman, T.D. Localized orbital/local origin method for calculation and analysis of NMR shieldings. Applications to ^{13}C shielding tensors. *J. Chem. Phys.* **1985**, *82*, 5035–5047.

(16) Komorovský, S.; Repiský, M.; Malkina, O. L.; Malkin, V. G.; Malkin Ondík, I.; Kaupp, M. A Fully Relativistic Method for Calculation of Nuclear Magnetic Shielding Tensors with a Restricted Magnetically Balanced Basis in the Framework of the Matrix Dirac–Kohn–Sham Equation. *J. Chem. Phys.* **2008**, *128*.

(17) Malkin, V. G.; Malkina, O. L.; Reviakine, R.; Arbuznikov, A. V.; Kaupp, M.; Schimmelpfennig, B.; Malkin, I.; Repisky, M.; Komorovsky, S.; Hrobarik, P.; Malkin, E.; Helgaker, T.; Ruud, K. MAG-ReSpect, version 5.1.0; 2019.

(18) Dyall, K. G. Relativistic Double-Zeta, Triple-Zeta, and Quadruple-Zeta Basis Sets for the 5d Elements Hf-Hg. *Theor. Chem. Acc.* **2004**, *112*, 403–409.

(19) Chesnut, D. B.; Moore, K. D. Locally Dense Basis Sets for Chemical Shift Calculations. *J. Comp. Chem.* **1989**, *10*, 648–659.

(20) Chesnut, D. B.; Rusiloski, B. E.; Moore, K. D.; Egolf, D. A. Use of Locally Dense Basis Sets for Nuclear Magnetic Resonance Shielding Calculations. *J. Comp. Chem.* **1993**, *14*, 1364–1375.

(21) Chesnut, D. B.; Byrd, E. F. C. The Use of Locally Dense Basis Sets in Correlated NMR Chemical Shielding Calculations. *Chem. Phys.* **1996**, *213*, 153–158.

(22) Provasi, P. F.; Aucar, G. A.; Sauer, S. P. A. The Use of Locally Dense Basis Sets in the Calculation of Indirect Nuclear Spin–Spin Coupling Constants: The Vicinal Coupling Constants in $H_3C–CH_2X$ (X=H, F, Cl, Br, I). *J. Chem. Phys.* **2000**, *112*, 6201–6208.

(23) Sanchez, M.; Provasi, P. F.; Aucar, G. A.; Sauer, S. P. A. On the Usage of Locally Dense Basis Sets in the Calculation of NMR Indirect Nuclear Spin–Spin Coupling Constants: Vicinal Fluorine–Fluorine Couplings. *Adv. Quantum Chem.* **2005**, *48*,161–183.

(24) Dunning, T. H., Jr. Gaussian basis sets for use in correlated molecular calculations. I. The atoms boron through neon and hydrogen. *J. Chem. Phys.* **1989**, *90*, 1007−1023.

(25) Woon, D. E.; Dunning, T. H. Gaussian basis sets for use in correlated molecular calculations. III. The atoms aluminum through argon. *J. Chem. Phys.* **1993**, *98*, 1358−1371.

(26) Wilson, A. K.; Woon, D. E.; Peterson, K. A.; Dunning, T. H., Jr. Gaussian Basis Sets for Use in Correlated Molecular Calculations. IX. The Atoms Gallium through Krypton. *J. Chem. Phys.* **1999**, *110*, 7667–7676.

(27) Jensen, F. Polarization Consistent Basis Sets: Principles. The Journal of Chemical Physics, 2001, 115, 9113–9125.

(28) Jensen, F. Polarization Consistent Basis Sets. II. Estimating the Kohn–Sham Basis Set Limit. The Journal of Chemical Physics, 2002, 116, 7372–7379.

(29) Jensen, F.; Helgaker, T. Polarization Consistent Basis Sets. V. The Elements Si–Cl. The Journal of Chemical Physics, 2004, 121, 3463–3470.

(30) Jensen, F. Polarization Consistent Basis Sets. VII. The Elements K, Ca, Ga, Ge, As, Se, Br, and Kr. The Journal of Chemical Physics, 2012, 136.

(31) Pritchard, B.P.; Altarawy, D.; Didier, B.; Gibson, T.D.; Windus, T.L. A New Basis Set Exchange: An Open, Up-to-date Resource for the Molecular Sciences Community. *J. Chem. Inf. Model.* **2019**, *59*, 4814–4820.

(32) Feller, D. The Role of Databases in Support of Computational Chemistry Calculations. *J. Comp. Chem.* **1996**, *17*, 1571–1586.

(33) Schuchardt, K. L.; Didier, B. T.; Elsethagen, T.; Sun, L.; Gurumoorthi, V.; Chase, J.; Li, J.; Windus, T. L. Basis Set Exchange: A Community Database for Computational Sciences. *J. Chem. Inf. Model.* **2007**, *47*, 1045–1052.

(34) Wicht, D. K.; Paisner, S. N.; Lew, B. M.; Glueck, D. S.; Yap, G. P. A.; Liable-Sands, L. M.; Rheingold, A. L.; Haar, C. M.; Nolan, S. P. Terminal Platinum(II) Phosphido Complexes: Synthesis, Structure, and Thermochemistry. *Organometallics*, **1998**, *17*, 652–660. https://doi.org/10.1021/om9708891.

(35) Song, D.; Wang, S. Benzene C−H Activation by Two Isomeric Platinum(II) Complexes of Bis(N-7-Azaindolyl)Methane. *Organometallics*, **2003**, *22*, 2187–2189. https://doi.org/10.1021/om0301785.

(36) Clark, H. C.; Ward, J. E. H. ^{13}C Nuclear Magnetic Resonance Studies of Organometallic Compounds. III. Cis-Methylplatinum(II) Derivatives. *Canadian Journal of Chemistry*, **1974**, *52*, 570–578. https://doi.org/10.1139/v74-089.

(37) Suslick, B. A.; Liberman-Martin, A. L.; Wambach, T. C.; Tilley, T. D. Olefin Hydroarylation Catalyzed by (Pyridyl-Indolate)Pt(II) Complexes: Catalytic Efficiencies and Mechanistic Aspects. *ACS Catalysis*, **2017**, *7*, 4313–4322. https://doi.org/10.1021/acscatal.7b01560.

(38) Mukhopadhyay, S.; Lasri, J.; Guedes da Silva, M. F. C.; Januário Charmier, M. A.; Pombeiro, A. J. L. Activation of C–CN Bond of Propionitrile: An Alternative Route to the Syntheses of 5-Substituted-1H-Tetrazoles and Dicyano-Platinum(II) Species. *Polyhedron*, **2008**, *27*, 2883–2888. https://doi.org/10.1016/j.poly.2008.06.031.

(39) Fujita, M.; Kim, W. H.; Sakanishi, Y.; Fujiwara, K.; Hirayama, S.; Okuyama, T.; Ohki, Y.; Tatsumi, K.; Yoshioka, Y. Elimination−Addition Mechanism for Nucleophilic Substitution Reaction of Cyclohexenyl Iodonium Salts and Regioselectivity of Nucleophilic Addition to the Cyclohexyne Intermediate. *Journal of the American Chemical Society*, **2004**, *126*, 7548–7558. https://doi.org/10.1021/ja0496672.

(40) Riera, X.; López, C.; Caubet, A.; Moreno, V.; Solans, X.; Font-Bardia, M. Platinum(II) and Palladium(II) Compounds Containing Chiral Thioimines. *European Journal of Inorganic Chemistry*, **2001**, *2001*, 2135–2141. https://doi.org/10.1002/1099-0682(200108)2001:8<2135::aid-ejic2135>3.0.co;2-2.

(41) Fuertes, S.; Chueca, A. J.; Sicilia, V. Exploring the Transphobia Effect on Heteroleptic NHC Cycloplatinated Complexes. *Inorganic Chemistry*, **2015**, *54*, 9885–9895. https://doi.org/10.1021/acs.inorgchem.5b01655.

(42) Pawlak, T.; Munzarová, M. L.; Pazderski, L.; Marek, R. Validation of Relativistic DFT Approaches to the Calculation of NMR Chemical Shifts in Square-Planar Pt^{2+} and Au^{3+} Complexes. *Journal of Chemical Theory and Computation*, **2011**, *7*, 3909–3923. https://doi.org/10.1021/ct200366n.

(43) Pazderski, L.; Pawlak, T.; Sitkowski, J.; Kozerski, L.; Szłyk, E. ^{1}H, ^{13}C, ^{15}N and ^{195}Pt NMR Studies of Au(III) and Pt(II) Chloride Organometallics with 2-phenylpyridine. *Magnetic Resonance in Chemistry*, **2009**, *47*, 932–941. https://doi.org/10.1002/mrc.2491.

(44) Carroll, J.; Gagnier, J. P.; Garner, A. W.; Moots, J. G.; Pike, R. D.; Li, Y.; Huo, S. Reaction of N-Isopropyl-N-Phenyl-2,2′-Bipyridin-6-Amine with K_2PtCl_4: Selective C–H Bond Activation, C–N Bond Cleavage, and Selective Acylation. *Organometallics*, **2013**, *32*, 4828–4836. https://doi.org/10.1021/om400540y.

(45) Hoogervorst, W. J.; Elsevier, C. J.; Lutz, M.; Spek, A. L. New Cis- and Trans-Arylplatinum(II) Acetylide Compounds Containing a Bis(Imino)Aryl [NCN] Ligand. *Organometallics*, **2001**, *20*, 4437–4440. https://doi.org/10.1021/om010447w.

(46) Zhang, X.; Wright, A. M.; DeYonker, N. J.; Hollis, T. K.; Hammer, N. I.; Webster, C. E.; Valente, E. J. Synthesis, Air Stability, Photobleaching, and DFT Modeling of Blue Light Emitting Platinum CCC-N-Heterocyclic Carbene Pincer Complexes. *Organometallics*, **2012**, *31*, 1664–1672. https://doi.org/10.1021/om200687w.

(47) Jia, Y.-X.; Yang, X.-Y.; Tay, W. S.; Li, Y.; Pullarkat, S. A.; Xu, K.; Hirao, H.; Leung, P.-H. Computational and Carbon-13 NMR Studies of Pt–C Bonds in P–C–P Pincer Complexes. *Dalton Transactions*, **2016**, *45*, 2095–2101. https://doi.org/10.1039/c5dt02049b.

(48) Brendel, M.; Engelke, R.; Desai, V. G.; Rominger, F.; Hofmann, P. Synthesis and Reactivity of Platinum(II) Cis-Dialkyl, Cis-Alkyl Chloro, and Cis-Alkyl Hydrido Bis-N-Heterocyclic Carbene Chelate Complexes. *Organometallics*, **2015**, *34*, 2870–2878. https://doi.org/10.1021/acs.organomet.5b00204.

(49) Weiss, D. T.; Altmann, P. J.; Haslinger, S.; Jandl, C.; Pöthig, A.; Cokoja, M.; Kühn, F. E. Structural Diversity of Late Transition Metal Complexes with Flexible Tetra-NHC Ligands. *Dalton Transactions*, **2015**, *44*, 18329–18339. https://doi.org/10.1039/c5dt02386f.

(50) Lu, T.; Liu, Z.; Steren, C. A.; Fei, F.; Cook, T. M.; Chen, X.-T.; Xue, Z.-L. Synthesis, Structural Characterization and NMR Studies of Group 10 Metal Complexes with Macrocyclic Amine N-Heterocyclic Carbene Ligands. *Dalton Transactions*, **2018**, *47*, 4282–4292. https://doi.org/10.1039/c7dt04666a.

(51) Seyboldt, A.; Wucher, B.; Hohnstein, S.; Eichele, K.; Rominger, F.; Törnroos, K. W.; Kunz, D. Evidence for the Formation of Anionic Zerovalent Group 10 Complexes as Highly Reactive Intermediates. *Organometallics*, **2015**, *34*, 2717–2725. https://doi.org/10.1021/om500836m.

(52) Dahm, G.; Bailly, C.; Karmazin, L.; Bellemin-Laponnaz, S. Synthesis, Structural Characterization and in Vitro Anti-Cancer Activity of Functionalized N-Heterocyclic Carbene Platinum and Palladium Complexes. *Journal of Organometallic Chemistry*, **2015**, *794*, 115–124. https://doi.org/10.1016/j.jorganchem.2015.07.003.

(53) Muenzner, J. K.; Rehm, T.; Biersack, B.; Casini, A.; de Graaf, I. A. M.; Worawutputtapong, P.; Noor, A.; Kempe, R.; Brabec, V.; Kasparkova, J.; Schobert, R. Adjusting the DNA Interaction and Anticancer Activity of Pt(II) N-Heterocyclic Carbene Complexes by Steric Shielding of the Trans Leaving Group. *Journal of Medicinal Chemistry*, **2015**, *58*, 6283–6292. https://doi.org/10.1021/acs.jmedchem.5b00896.

(54) Bouché, M.; Dahm, G.; Wantz, M.; Fournel, S.; Achard, T.; Bellemin-Laponnaz, S. Platinum(IV) N-Heterocyclic Carbene Complexes: Their Synthesis, Characterisation and Cytotoxic Activity. *Dalton Transactions*, **2016**, *45*, 11362–11368. https://doi.org/10.1039/c6dt01846g.

(55) Crumpton-Bregel, D. M.; Goldberg, K. I. Mechanisms of C−C and C−H Alkane Reductive Eliminations from Octahedral Pt(IV): Reaction via Five-Coordinate Intermediates or Direct Elimination? *Journal of the American Chemical Society*, **2003**, *125*, 9442–9456. https://doi.org/10.1021/ja029140u.

(56) Scheuermann, M. L.; Luedtke, A. T.; Hanson, S. K.; Fekl, U.; Kaminsky, W.; Goldberg, K. I. Reactions of Five-Coordinate Platinum(IV) Complexes with Molecular Oxygen. *Organometallics*, **2013**, *32*, 4752–4758. https://doi.org/10.1021/om4003363.

(57) Green, M.; Howard, J. A. K.; Mitrprachachon, P.; Pfeffer, M.; Spencer, J. L.; Stone, F. G. A.; Woodward, P. Organo-Complexes of Platinum Derived from Methyl Vinyl Ketone and Bis(Cyclo-Octa-1,5-Diene)Platinum; X-Ray Crystal Structure of (1,3-Diacetylbutane-1,4-Diyl)Bis(Triphenylphosphine)Platinum. Journal of the Chemical Society, *Dalton Transactions*, **1979**, *306*. https://doi.org/10.1039/dt9790000306.

(58) Ogoshi, S.; Morita, M.; Kurosawa, H. Synthesis, Structure, and Reactivity of a η^3-1-Hydroxyallyl Complex: Protonation of an α,β-Unsaturated Carbonyl Compound Bound to Palladium(0) and Platinum(0). *Journal of the American Chemical Society*, **2003**, *125*, 9020–9021. https://doi.org/10.1021/ja0361042.

(59) Colebatch, A. L.; Cade, I. A.; Hill, A. F.; Bhadbhade, M. M. η^2-Allenyl- and η^2-Alkynylphosphonium Complexes of Platinum. *Organometallics*, **2013**, *32*, 4766–4774. https://doi.org/10.1021/om400406s.
